# Polypeptide Preparation by β-Lactone-Mediated Chemical Ligation

**DOI:** 10.1021/acs.orglett.4c01587

**Published:** 2024-06-20

**Authors:** Xinhao Fan, Yuming Wen, Huan Chen, Baotong Tian, Qiang Zhang

**Affiliations:** † Department of Chemistry, School of Pharmacy, North Sichuan Medical College, Nanchong, Sichuan 637000, China; ‡ Department of Chemistry, University at Albany, State University of New York, 1400 Washington Avenue, Albany, NY 12222, USA

## Abstract

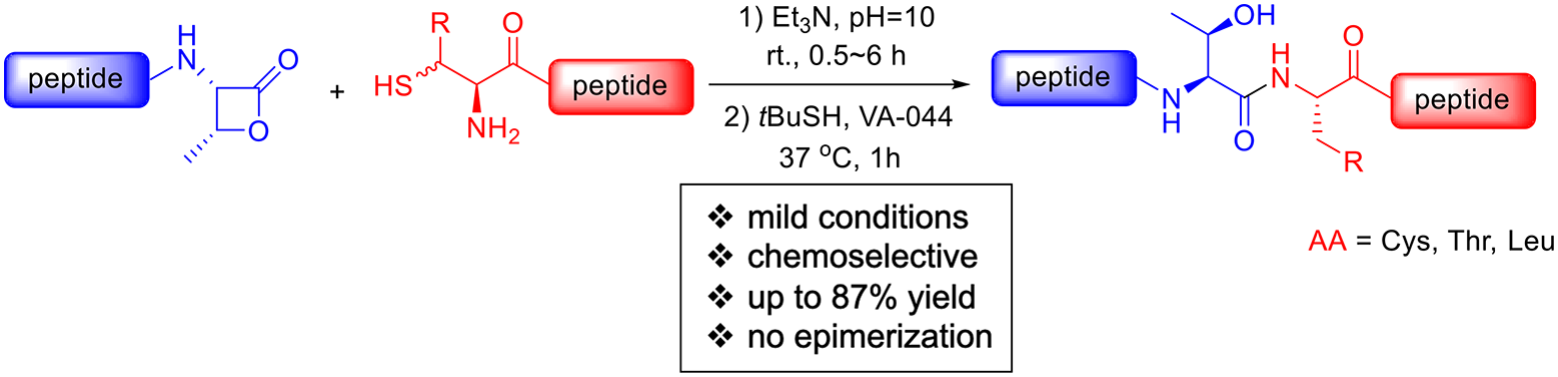

Native chemical ligation (NCL) represents a cornerstone strategy in accessing synthetic peptides and proteins, remaining one of the most efficacious methodologies in this domain. The fundamental requisites for achieving a proficient NCL reaction involve chemoselective coupling between a C-terminal thioester peptide and a thiol-bearing N-terminal peptide. However, achieving coupling at sterically congested residues remains challenging. In addition, while most NCLs proceed without epimerization, β-branched (e.g., Ile, Thr, Val) and Pro-derived C-terminal thioesters react slowly and can be susceptible to significant epimerization and hydrolysis. Herein, we report an epimerization-free NCL reaction via β-lactone-mediated native chemical ligation which constructs sterically congested Thr residues. The constrained ring from the β-lactone allows rapid peptide ligation without detectable epimerization. The method has a broad side-chain tolerance and was applied to the preparation of cyclic peptides and polypeptidyl thioester, which could be difficult to obtained otherwise.

## Introduction

**Figure 1:**
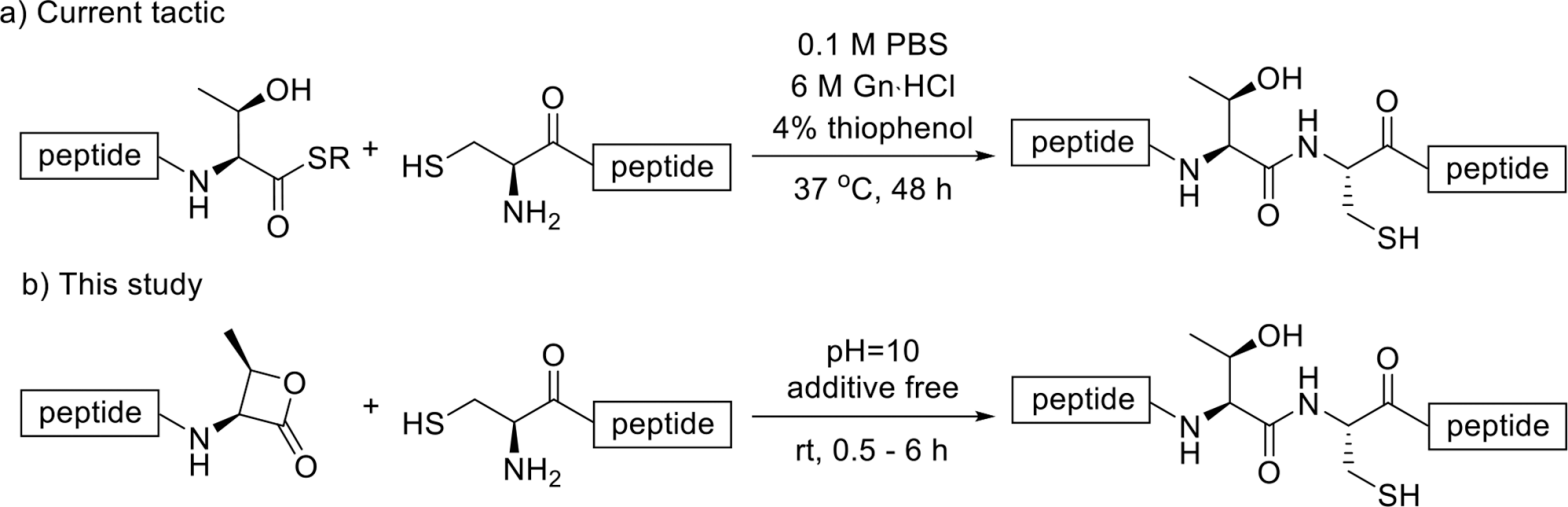
β-Lactone-mediated native chemical ligation.

The application of native chemical ligation (NCL) in chemical protein preparation has yielded considerable success, facilitating the synthesis of a wide range of biologics, including membrane proteins and glycoproteins.^1−7^ NCL not only aids in native protein assembly but also efficiently enables the creation of peptide and protein analogues containing noncanonical residues.^8^ The cornerstone of successful NCL lies in the incorporation of a C-terminal thioester as an acyl donor, which enables chemoselective transthioesterification between two peptidyl fragments. One significant challenge encountered during peptide transthioesterification is the linking of amino acid residues with steric hindrance. Amino acid residues featuring bulky side chains at the reacting C-terminal thioester demand extended reaction periods, often accompanied by low yields and epimerization, thereby constraining the efficiency and outcomes of the ligations. The thioesters derived from Pro, Val, Ile, Leu, and Thr are particularly difficult to ligate.^9−11^ For example, it was reported that the ligation between a Thr thioester and Cys residue requires more than 48 h to complete^12^ (Figure 1a). In addition, Ser residues have been reported to incur substantial epimerization.^13^ In search of more capable peptidyl C-terminal preactivation, more effective thioesters to assist NCL have been extensively investigated.^9,14,15^ Among all proteinogenic amino acid residues, the occurrences of Thr-Cys and Thr-Ala connections in protein peptide bonds are 5.57% and 6.02%, respectively.^16^ Therefore, developing a new methodology to enable Thr-Xaa junctions is crucial. We present a distinct C-terminal activator derived from Thr residues: a strained β-lactone moiety. The efficiency and practicality of β-lactone-mediated ligations were demonstrated as a one-pot ligation method at the Thr site and significantly accelerated the NCL reaction time without epimerization (Figure 1b).

## Results and Discussion

**Scheme 1:**
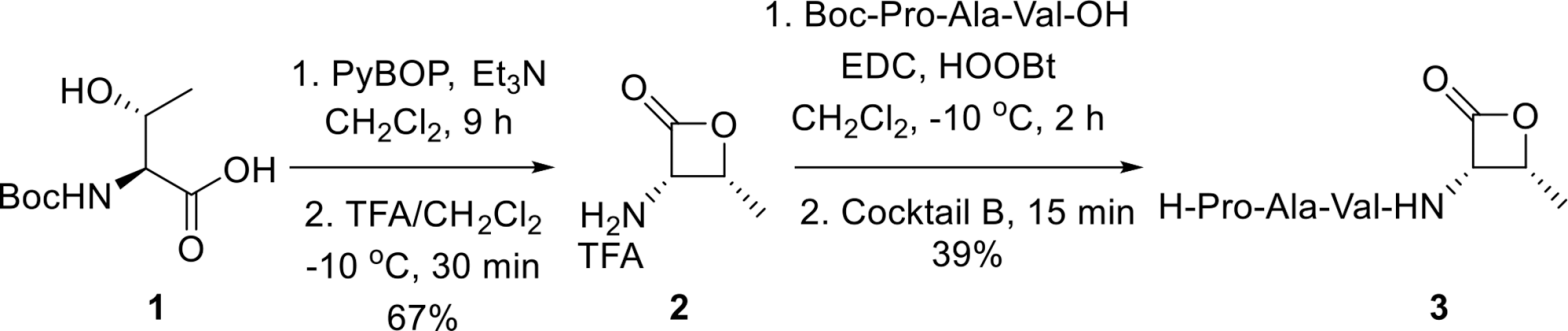
Synthesis of Polypeptide Containing C-Terminal β-Lactone

In a previous study, we illustrated the employment of constrained moieties in the form of β-thiolactones as thioester surrogates. This approach effectively addresses the ligation hurdle encountered at the C-terminal valine residue, facilitating the swift connection of two peptidyl fragments through one-pot ligation and subsequent desulfurization (Val-Xxx). Releasing ring constraints not only markedly accelerates ligation rates but also permits the linking of sterically hindered amino acid residues at ligation sites. However, converting residues such as Thr to β-thiolactone scaffolds is not viable without eroding the Thr side chain stereochemistry. Subsequently, we opt to utilize the analogous Thr β-lactone as a C-terminal activator for desired NCL construction.^17−19^ Investigation began with the synthesis of polypeptides bearing a Thr-derived β-lactone from commercially available amino acid (Scheme 1).^20^ β-Lactone TFA salt **2** could be produced after two steps in high yield (67%) from Boc-l-threonine **1**. Installation of C-terminal activated polypeptide **3** was accomplished by coupling **2** with Boc-Pro-Ala-Val-OH under EDC/HOOBt conditions,^21^ then subsequent global deprotection followed by HPLC purification furnished peptide **3** in decent yield over two steps (39%).

The initial assessment of chemical ligation between peptide **3** and cysteine-bearing peptide **4** was conducted in PBS buffer (Table 1). At pH = 7.8, only hydrolysis of β-lactone can be found (**6**). We suspected that reducing the solution pH might render ligated product formation. Unfortunately, decreasing the pH to 6.8 furnished hydrolysis product **6** exclusively. The attempt to use a water-soluble organic base (Et_3_N, pH 8, entry 3) as the reaction medium failed to generate any product. It is evident that the aqueous solution outcompetes the desired thiolysis, rapidly opening the β-lactone and yielding Thr at the C-terminus. Based on literature, cysteine thiolate is 10,000 times more nucleophilic than other amino acid residues in basic buffer.^22,23^ We speculate that at elevated pH levels, thiolate may rival solvolysis. Subsequently, increase reaction pH to 10 (Et_3_N, entry 4) furnished the desired ligation, and product **5** was generated in 82% yield, whereas byproduct **6** was observed in less than 5%. Although Et_3_N is commonly regarded as non-nucleophilic, we conducted experiments to eliminate the possibility of β-lactone ring opening by Et_3_N before transthioesterification. Ligations conducted under basic conditions using NaOH yielded similar results (entry 5), while attempts with DABCO (entry 6) and imidazole (entry 7)^24^ did not yield the desired product at the same pH. We suspected that the (albeit marginal) increased nucleophilicity of DABCO and imidazole might impede transthioesterification through nucleophilic ring opening of the Thr β-lactone. However, no ring-opening products derived from DABCO or imidazole were detected during the reaction. Ultimately, we observed that increasing the temperature did not enhance ligation yield (entry 8) and reducing the pH to 8 in the presence of Et_3_N failed to yield the desired product (entry 3). Based on the observed data, at a higher pH level (∼10), the formation of cysteine thiolate (SH → S^–^) facilitates the opening of the β-lactone ring, thus promoting ligation. However, the success of this ligation is not solely dependent on the reaction pH. The presence of nucleophilic additives in solution can disrupt the desired ligation process and result in hydrolysis.

**Table 1:**
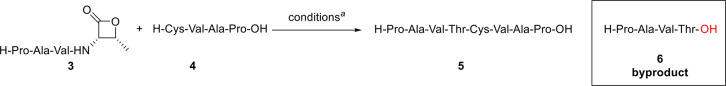
β-Lactone Mediated Native Chemical Ligation

Entry	Condition	Yield of **5**	Yield of **6**
1	PBS buffer pH = 7.8	none	89%
2	PBS buffer pH = 6.8	none	84%
3	Et_3_N pH = 8	none	88%
4	Et_3_N pH = 10	82%	<5%
5	NaOH pH = 10	68%	16%
6	DABCO pH = 10	none	78%
7	imidazole pH = 10	none	75%
8^b^	Et_3_N pH = 10	71%	15%

a Reaction conditions: 0.1 eq of **5** mixed with 1 eq of **6** in different solutions to make the concentration 10 mM at 25 °C in 1 h.

b Same ligation parameters at 35 °C in 0.5 h.

With optimized reaction conditions in hand (Et_3_N, pH = 8 in water), the reaction scope was explored (Table 2). The β-lactone-incorporated peptides were synthesized according to the method outlined in Scheme 1, and C-terminal epimerization was not detected during the preparation of polypeptide **7**. The coupling of peptide **3** with cysteine-bearing peptides **9** and **11** resulted in the production of polypeptides **10** and **12** in excellent yields (89% and 93%, respectively). Subsequently, our methods facilitated the successful coupling of more intricate polypeptide sequences, resulting in ligated products with consistently high yields (entries 3–5). Furthermore, we observed that the 4-mercapto-threonine-bearing peptide **20** successfully enabled ligation at Thr-Thr when reacted with β-lactone peptide **3**, followed by metal-free desulfurization, yielding a good overall yield of 58% after two steps (entry 6). An identical protocol was employed for the one-pot synthesis of the Thr-Leu linkage between octapeptides **22** and **24**, resulting in a yield of 38% over two steps. The fair reaction yield was due to the low-efficiency desulfurization step, which hampered the overall outcome. In general, reactive residues, such as Lys, Asp, Thr, His, Arg, Tyr, Met, and Ser, do not interfere with the ligation process. Beyond conventional NCL, β-lactone-mediated ligation offers a method for connecting noncanonical peptides. For instance, the ligation between a β-lactone-bearing peptide **26** and a non-natural peptide **27** (with 2-mercaptoacetic acid at the N-terminus) successfully produced thioester 29-mer **28** in good yield (entry 9). These findings highlight the potential utility of the C-terminal β-lactone in peptide chemical synthesis. Although serine-derived β-lactone was initially considered as a candidate for C-terminal activation, it proved to be too labile for ligation. The compound decomposed during the EDC coupling step and could not be used in polypeptide synthesis. Thiolysis exclusively took place between the carbonyl of β-lactone (C1) and the cysteine thiol group. No ring opening at C3 was observed,^25^ likely due to the steric hindrance of the methyl group.

**Table 2:**
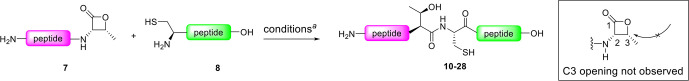

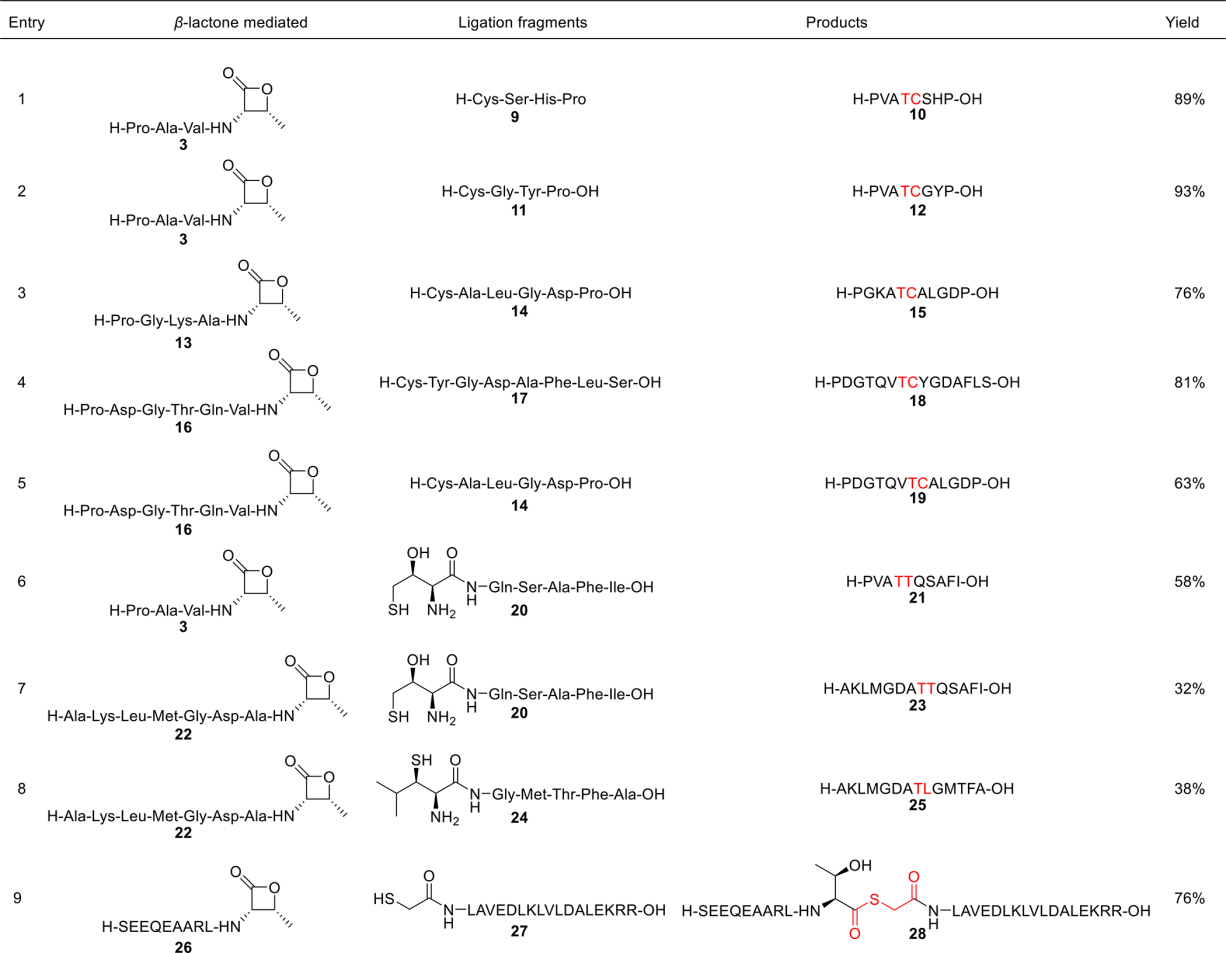
Scope Study of β-Lactone-Mediated Native Chemical Ligation

a Reaction conditions: pH =10, Et_3_N, H_2_O, rt, 30 min to 6 h.

**Figure 2:**
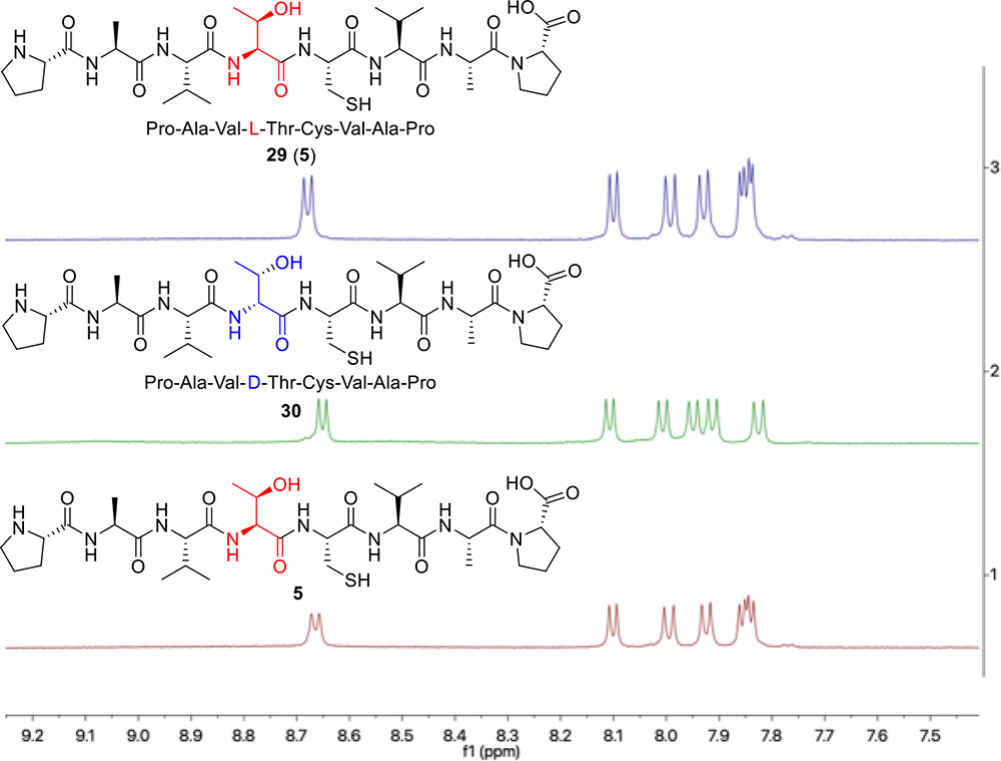
^1^H NMR spectra of ligation product l- and d-isomers.

The activation of the C-terminus may induce epimerization at the α-center. We conducted an evaluation of potential epimerization at the Thr ligation site, considering the constraints and reactivity associated with β-lactone. Pro-Ala-Val-(l)-Thr-Cys-Val-Ala-Pro **29** (**5**) and its isomer Pro-Ala-Val-(d)-Thr-Cys-Val-Ala-Pro **30** was directly prepared in parallel by Solid Phase Peptide Synthesis (SPPS). The comparative ^1^H NMR spectrum data indicated ligation product **5** is consistent with the spectrum of synthetic Pro-Ala-Val-(l)-Thr-Cys-Val-Ala-Pro **29** (**5**), whereas the ^1^H spectrum of Pro-Ala-Val-(d)-Thr-Cys-Val-Ala-Pro **30** demonstrated distinct peaks with chemical shifts around 7–8 ppm (Figure 2). NMR spectroscopy experiments suggested that no detectable epimerization occurred during the β-lactone-mediated ligation. This may be attributable to the near-flat geometry of the β-lactone moiety, which is unlikely to permit enolization and subsequent stereocenter erosion.^26^

**Scheme 2:**
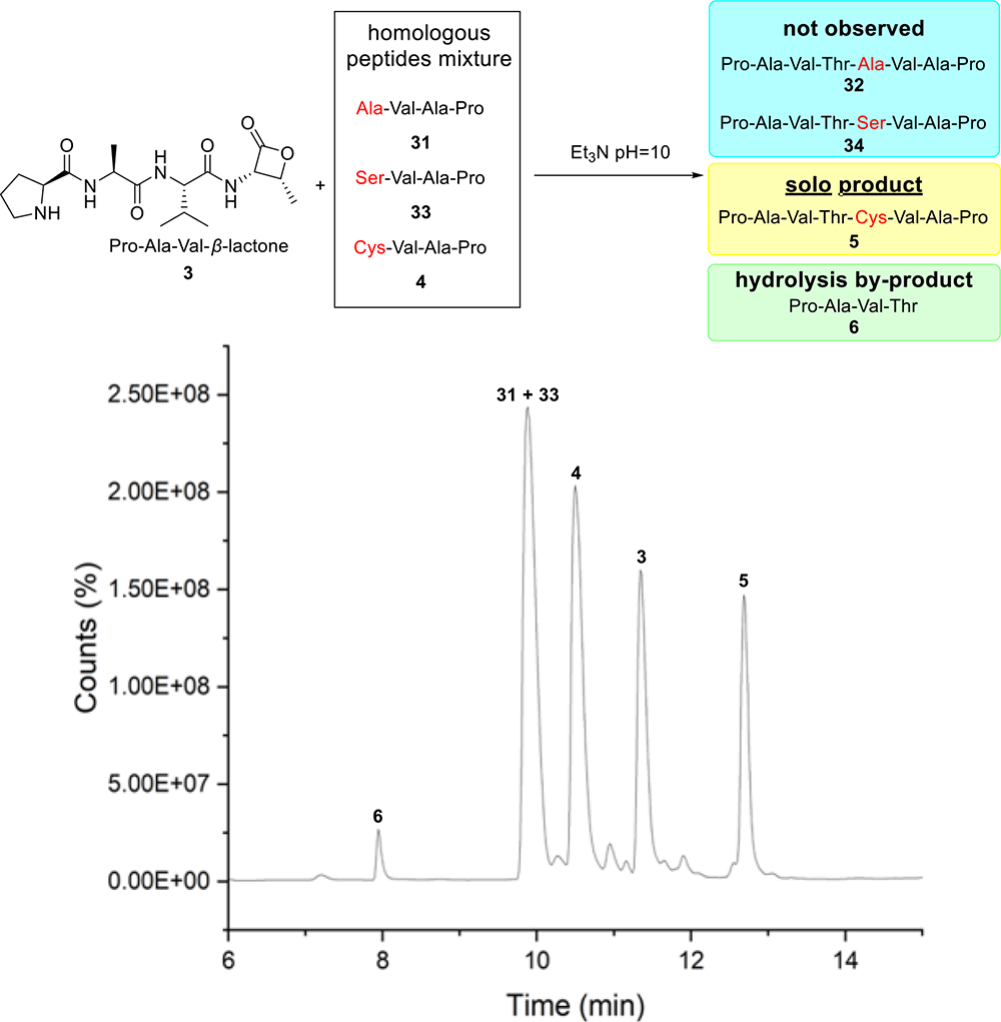
Competition Study of β-Lactone-Mediated Native Chemical Ligation

To confirm the chemoselectivity of the C-terminal β-lactone, a competition experiment was devised. Alongside the desired intermolecular thiolysis, competing hydrolysis^15^ and aminolysis^27^ processes could potentially take place. Tetrapeptides Ala-Val-Ala-Pro **31** and Ser-Val-Ala-Pro **33** (analogue of **4**) were obtained. The experiment was carried out by mixing β-lactone peptide **3** with three different scaffolds bearing N-terminal Ala (**31**), Ser (**33**), and Cys (**4**) under standard conditions, which may generate three distinct ligated peptides **32**, **34**, and **5** (Scheme 2). After 10 min in the aqueous solution (Et_3_N, pH = 10), the LCMS trace of the crude reaction suggested the formation of exclusive Cys ligation product **5** at 12.5 min, which suggested the chemoselectivity between Thr β-lactone and Cys residues. No intermolecular hydrolysis or aminolysis products were detected, and the only additional substrate observed in LC was a small fraction of the β-lactone hydrolysis product **6**.

**Scheme 3:**
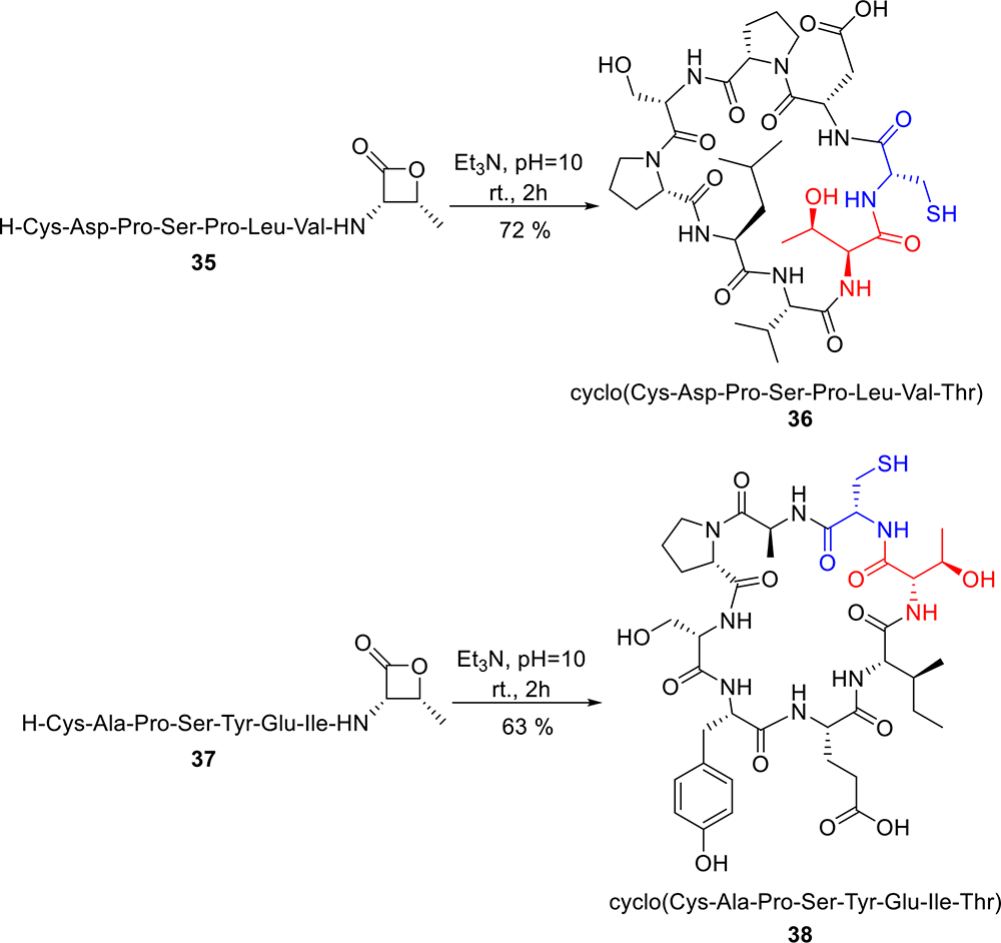
Synthesis of Cyclic Peptides

Utilizing the developed methodology, we showcased the applicability of β-lactone-mediated peptide ligation by preparing cyclic peptides (Scheme 3). Linear peptides **35** and **37** were obtained, and intramolecular ring closure of **34** and **36** was achieved under optimized conditions. Within 2 h, the corresponding cyclic peptides **36** and **38** were obtained in good yields (72% and 63% respectively).

## Conclusion

In summary, we introduced a novel method for C-terminal activation in peptide ligation. The β-lactone ring strain release overcomes residue steric hurdles and enables shorter ligation times (30 min to 6 h). The thiolysis of β-lactone is completely chemoselective to generate peptidyl bonds. This protocol allows Thr-Xaa ligation sites after one-pot ligation and desulfurization with broad residue tolerance. β-Lactone-mediated ligation can be utilized for both intermolecular and intramolecular peptide ligations, allowing for the rapid synthesis of elongated polypeptides and cyclic peptides. Lastly, β-lactone chemistry offers a solution for coupling at sterically congested Thr residues without epimerization and facilitates the efficient introduction of noncanonical residues into the peptidyl backbone, such as midsequence peptide thioesters.

## Data Availability

The data underlying this study are available in the published article and its Supporting Information.

## Supporting Information Available

Complete experimental procedures and characterization data for all new compounds (PDF)
