# Supplementary material for: Polypeptide Preparation by β-Lactone-Mediated Chemical Ligation
Source: Org Lett. 2024 Jun 20;26(26):5436–40. doi: 10.1021/acs.orglett.4c01587 (PMC11232016; doi:10.1021/acs.orglett.4c01587)
Supplement: Supplementary file 1 — ol4c01587_si_001.pdf [file ol4c01587_si_001.pdf]

Supporting Information

Polypeptide preparation by β-lactone-mediated chemical ligation

Xinhao Fan,[1,2] Yuming Wen,[2] Huan Chen,[2] Baotong Tian,[2] and Qiang Zhang[2]*

1. Department of Chemistry, School of Pharmacy, North Sichuan Medical College, Nanchong, Sichuan 637000, China

2. Department of Chemistry, University at Albany, State University of New York, 1400 Washington Avenue, Albany, NY 12222 (USA).

*Correspondence to: qzhang5@albany.edu,

Table of Contents

I General Information **2**

II Procedures for β-lactone Synthesis **2**

III Procedures for β-lactone Mediated Peptide Synthesis **4**

IV Preparation and Characterization of Peptide **6**

V Epimerization Free Experiment **35**

VI Four-component NCL Competition Experiment **37**

VII Cyclic Peptides Synthesis **38**

VIII Reference: **42**

I General Information

^{1}H NMR spectra were recorded at 500 MHz at ambient temperature with Dimethyl sulfoxide-d6 (Cambridge Isotope Laboratories, Inc.) as the solvent unless otherwise stated. Chemical shifts are reported in parts per million relative to DMSO-d6 (^{1}H, δ 2.5). Analytical High-Performance Liquid Chromatography (HPLC) and Liquid chromatography-low resolution mass spectrometry (LC-LRMS) were performed using a Waters® 2896 Photodiode array detector and Waters® SQ detector 2 with ZSpray™ source architecture single quadrupole detection system equipped with Waters® e2695 separation module. Analytical thin layer chromatography was performed using 0.25 mm silica gel 60-F plates. Flash chromatography was performed using 200-400 mesh silica gel. (Scientific Absorbents, Inc.) Yields refer to chromatographically and spectroscopically pure materials unless otherwise stated. All other reagents were purchased from Sigma-Aldrich, Alfa Aesar, Chemimpex and Oakwood Chemicals. All reactions were carried out in oven-dried glassware under an argon atmosphere unless otherwise noted. All commercially available materials (Aldrich®, Novabiochem®) were used without further purification. 2,2'-Azobis[2-(2-imidazolin-2-yl) propane] dihydrochloride (VA-044) was purchased from Wako Pure Chemical Industries. HATU was purchased from Genscript® (Piscataway, New Jersey). All reactions were performed under an atmosphere of pre-purified dry Ar (g). Analytical TLC was performed on SiliCycle silica gel 60 F254 plates and flash column chromatography was performed on SiliCycle silica gel 60 (40–63 mm). Yields refer to chromatographically pure compounds. Optical rotations were reported as follows: $[\alpha]_D^{20}$ (c: g/100 mL, in dichloromethane or chloroform).

HPLC: All separations involved a mobile phase of 0.05% TFA (*v*/*v*) in water (solvent A)/acetonitrile (solvent B). Analytical LC-MS analyses were performed using a Waters 2695 Separations Module and an Agilent G6530BA Q-TOF Mass Spectrometer equipped with Proto Microsorb 200, C18 150 × 2.0 mm, and Waters Microsorb 300-5, C4 250 x 2.0 mm columns at a flow rate of 0.5 mL/min. Preparative separations were performed using a Dionex Ultimate UHPLC system equipped with a UV detector and Proto reverse phase HPLC column Microsorb 200 C18 (250 × 20 mm) or a Microsorb 300-5 C4 (250 × 20 mm) at a flow rate of 4.0 mL/min.

II Procedures for β-lactone Synthesis

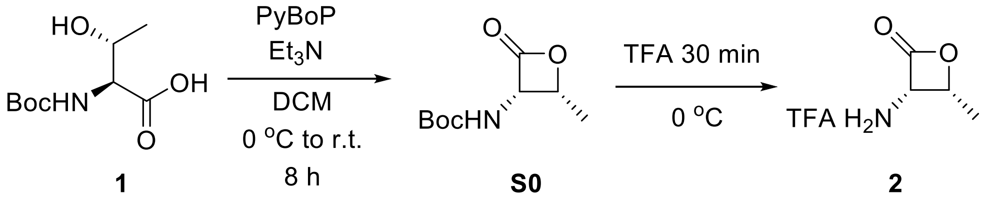

To a solution of Boc-L-Thr-OH (**1**) (1.10 g, 5 mmol) in dry DCM (100 mL) was added PyBOP at 0 °C, and trimethylamine (2.09 mL, 15 mmol, 3 equiv.) was added dropwise. The resulting mixture was raised to room temperature in 1 hour. After stirring for 8 hours, the reaction mixture was concentrated in *vacuo*. The residue was purified by silica gel chromatography (hexane/ EtOAc = 6: 1) to afford 672 mg (13.34 mmol, 67%) of (3S,4R)-3-[(tert-butyloxycarbonyl)amino]-4-methyloxetan-2-one (**S0**) as a white solid (M.p. = 138-140 °C, $[\alpha]_D^{20}$= 21.78° in CH_2Cl_2). The NMR spectra of **S0** was identical to the data as reported in the literature. [1]

^{1}H NMR (500 MHz, Chloroform-*d*) δ 5.47 – 5.38 (m, 1H), 5.20 (s, 1H), 4.88 – 4.82 (m, 1H), 1.46 (s, 12H).

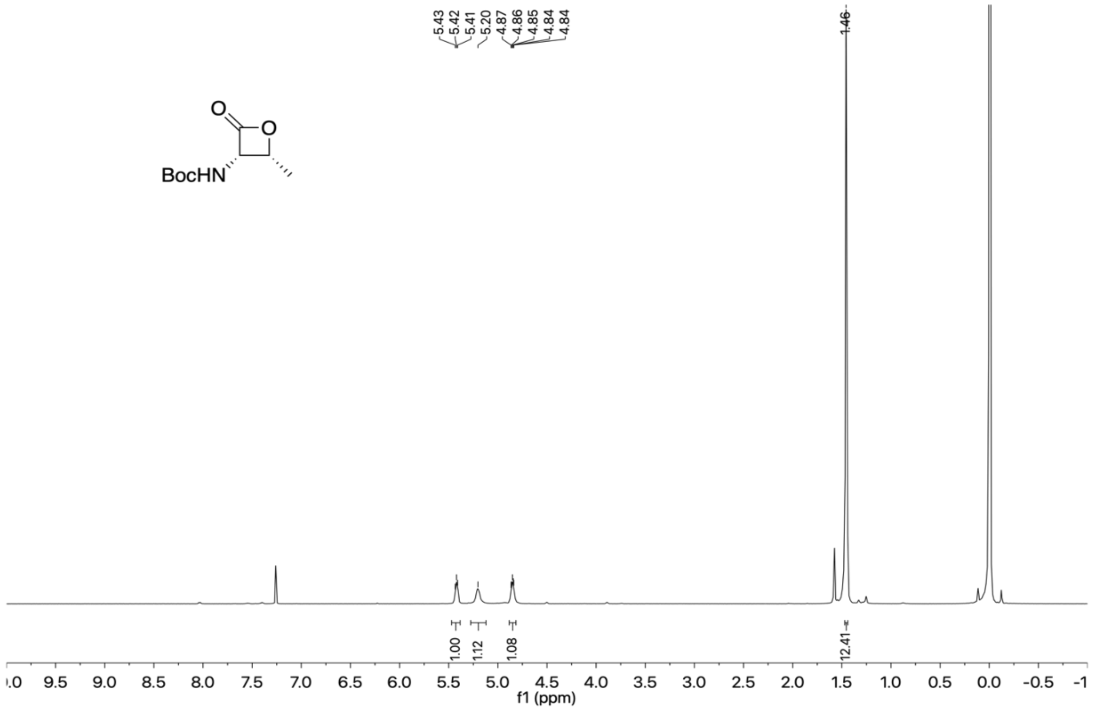

S0 (200 mg) was dissolved in trifluoroacetic acid (TFA) under ice bath. After stirring for 30 min, TFA was blown away by air flow. The residual TFA was removed by lyophilizer to yield 180.2 mg (3S,4R)-3-amino-4-methyloxetan-2-one TFA salt (**2**) (crude yield = 92%, $[\alpha]_D^{20}$= 5.39° in $CHCl_3$). The crude residual was used in the next step directly.

^{1}H NMR (500 MHz, Chloroform-d) δ 5.25 (d, *J* = 6.1 Hz, 1H), 5.15 (p, *J* = 6.3 Hz, 1H), 1.71 (d, *J* = 6.5 Hz, 3H).

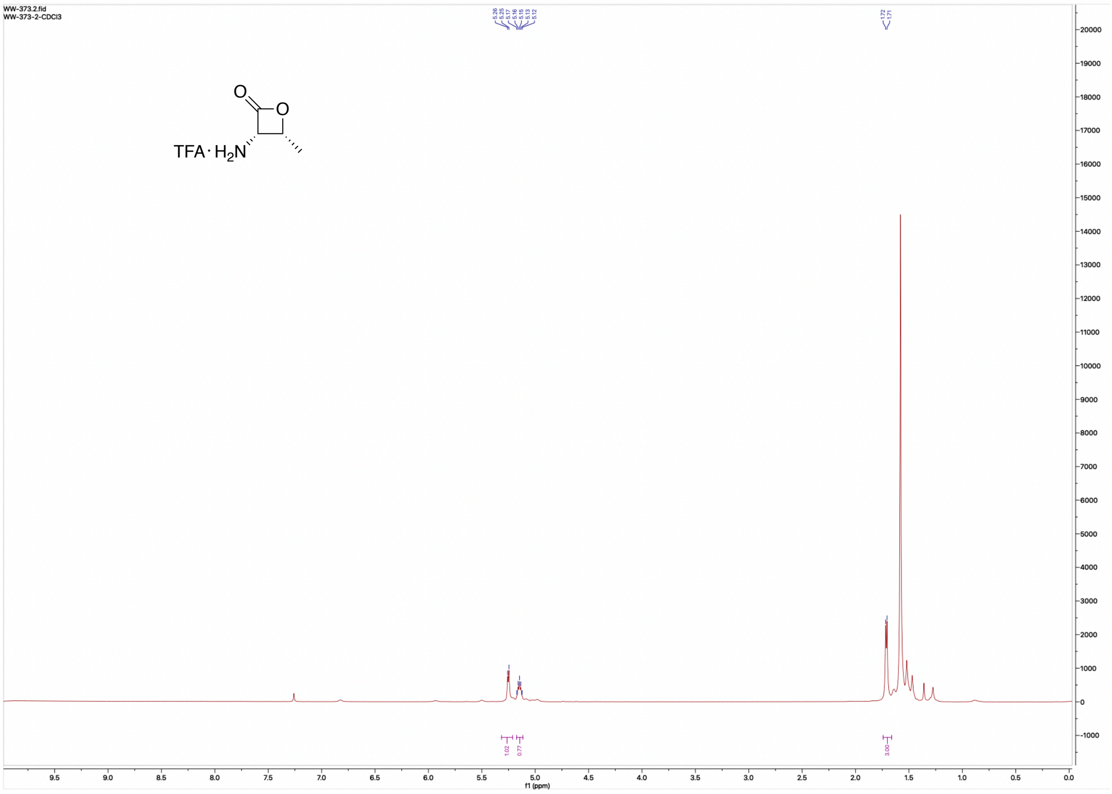

III Procedures for β-lactone Mediated Peptide Synthesis

A. Automated solid-phase peptide synthesis

Automated Solid-Phase peptide synthesis (SPPS) was performed on a Biotage peptide synthesis system (Initiator+ Alstra).

The Fmoc-Xxx-NovaSyn® TGT resin was employed in SPPS. Peptides were synthesized under standard automated Fmoc protocols using DMF as solvent, deblocking for 10 min in piperidine/ DBU/ DMF (2: 2: 96, V/ V/ V), coupling with HATU as coupling reagent for 25 min.

The following $^{\alpha}$*N*-Fmoc or $^{\alpha}$*N*-Boc-protected amino acids from Novabiochem or Chem-impex were employed in SPPS: Fmoc-Ala-OH, Fmoc-Arg(Pbf)-OH, Fmoc-Asn(Trt)-OH, Fmoc-Asp(O*t*Bu)-OH, Fmoc-Glu(O*t*Bu)-OH, Fmoc-Gln(Trt)-OH, Fmoc-Gly-OH, Fmoc-His(Trt)-OH, Fmoc-Ile-OH, Fmoc-Leu-OH, Fmoc-Lys(NHBoc)-OH, Fmoc-Met-OH, Fmoc-Phe-OH, Fmoc-Pro-OH, Fmoc-Ser(*t*Bu)-OH, Fmoc-Thr(*t*Bu)-OH, Fmoc-Trp(Boc)-OH, Fmoc-Tyr-OH, Fmoc-Val-OH, Boc-Glu(O*t*Bu)-OH, Boc-Cys(S*t*Bu)-OH, Boc-Cys(Trt)-OH, Boc-Ser(O*t*Bu)-OH.

B. Preparation of regular polypeptides

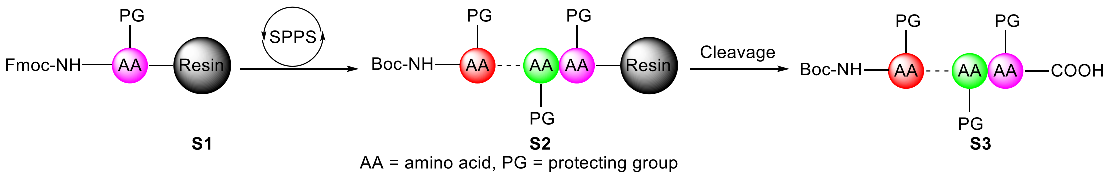

Scheme S1 Preparation of polypeptides by SPPS.

Upon completion of the automated synthesis on a 0.08 mmol scale, the peptide resin was washed into a peptide synthesis vessel by using 3×5 mL dichloromethane. The resin was cleaved by CH_2Cl_2/ TFE/ AcOH (8: 1: 1, *v* / *v* / *v*) solution for 20 min (× 2). After washing and filtration, the combined cleavage solution was concentrated under reduced pressure. The remaining residue was dissolved in a mixture of acetonitrile and water (*v* / *v* = 1 / 1), which was lyophilized to remove the solvent. Without further purification, the lyophilized solid was directly used in the next coupling step.

C. Preparation of *β*-lactone mediated peptides

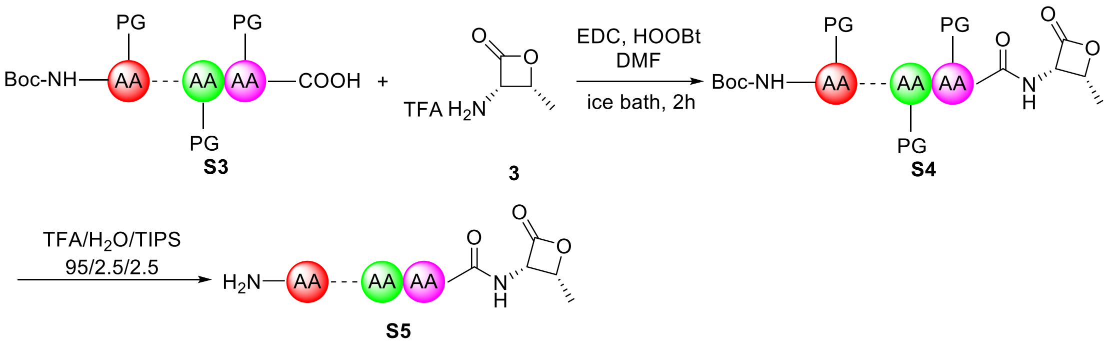

Scheme S2 Preparation of β-lactone mediated peptides.

Sidechain fully protected peptide was prepared according to general procedure **A** and **B**. To the mixture of **S3** (1.0 equiv.), the β-lactone TFA salt **2**(2 equiv.) and 3-hydroxy-1,2,3-benzotriazin-4-one (HOOBt) (2 equiv.) were added anhydrous DMF. The resulting solution was stirred at 0 °C for 2 min, and *N*-(3-Dimethylaminopropyl)-*N*-ethylcarbodiimide (EDC) (2 equiv.) was added. The resulting mixture was stirred at 0 °C for another 2 h. The reaction was quenched with saturated NH_4Cl and extracted with CH_2Cl_2 (5 mL × 3); the organic layer was dried over anhydrous Na_2SO_4. The Na_2SO_4 was filtered, and the solvent was removed *via* vacuum to afford a crude oily residue which was subjected to appropriate cocktail deprotection conditions at room temperature. After acid deprotection, the resulting solution was gently blown off by an argon stream to afford oily residue again. Finally, the oily residue was washed with cold diethyl ether to yield a white solid, which was dissolved in a mixture of acetonitrile and water and ready for HPLC purification after filtration.

D. Native chemical ligation

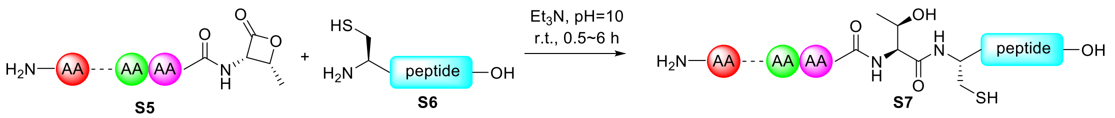

Scheme S3 Native chemical ligation.

To a mixture of the *β*-lactone mediated peptides **S6** (1.5 equiv.) and *N*-terminal mercapto-containing amino acid **S7** (1.0 equiv.) was added ligation solution (prepared

by adding Et_3N to DI water until the pH=10) under an argon atmosphere, and the resulting solution was stirred at room temperature and monitored by LC-MS. Upon completion, the reaction was quenched by adding H_2O/MeCN (90:10, *v*/*v*) and further purified by HPLC.

E. Metal-free desulfurization (MFD)
Prepare MFD Buffer:
Solution 1: 0.5 M TCEP, dissolve TCEP (50mg) in degassed water and adjust the pH to 7.2 with 10 M NaOH to a total volume of 300 μL.
To a mixture of thiol-containing peptide (2 mM) in 200 μL degassed solution 1 was added 50 μL of 2-methyl-2-propanethiol (*t*BuSH) and 75 μL of radical initiator VA-044 (0.1 M in degassed water). The reaction was stirred at 37 °C and monitored by LC-MS. Upon completion, the reaction was quenched by adding H_2O/MeCN (90:10, *v*/*v*) and further purified by HPLC.

IV Preparation and Characterization of Peptide

A. β-lactone mediated peptide

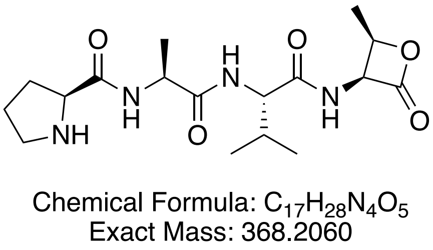

3

According to the general procedure A and B, the tripeptide Boc-Pro-Ala-Val-OH was generated directly from SPPS on a 0.08 mmol scale. The resulting tripeptide Boc-Pro-Ala-Val-OH was coupled with β-lactone TFA salt **2** to afford the desired tetrapeptide following the general procedure C. Purification of the crude product using preparative HPLC (10 to 50% solvent B over 20 min, Higgins Analytical Proto 200 5 μm 250 × 10 nm C18 column) afforded peptide **3** as a white solid after lyophilization (8.9 mg, 39%).

HPLC (Higgins Analytical Proto 200 5 μm 150 × 2.0 nm C18 column, water/acetonitrile = 90/10 to 40/60 over 20 min, flow rate = 0.5 mL/min, λ = 214 nm), tR= 11.13 min.

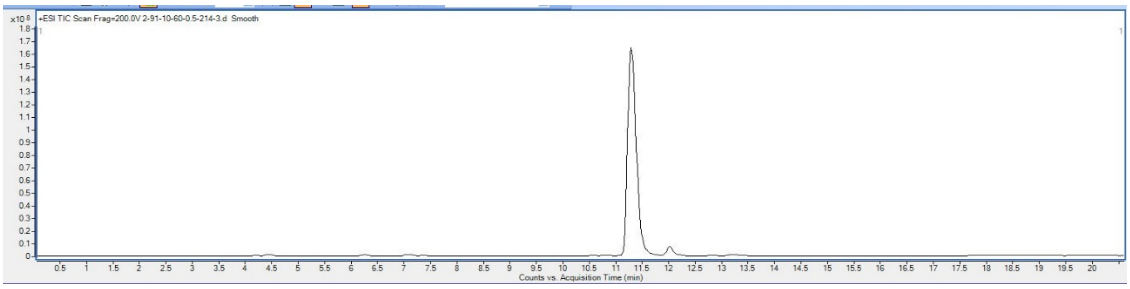

HRMS (ESI) m/z: $[M+H]^+$ Calcd for $C_{17}H_{28}N_4O_5$ 369.2138; Found 369.2169.

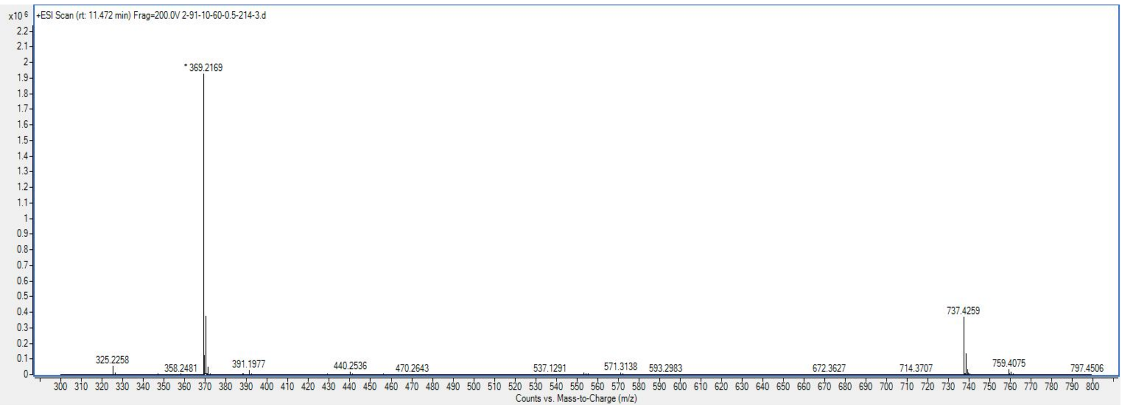

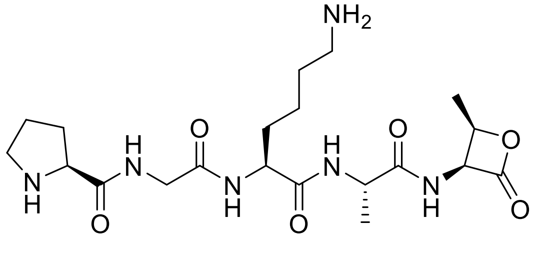

Chemical Formula: $C_{20}H_{34}N_6O_6$
Exact Mass: 454.2540
13

According to the general procedure A and B, the tetrapeptide Boc-Pro-Gly-Lys(*t*Bu)-Ala-OH was generated directly from SPPS on a 0.08 mmol scale. The resulting tetrapeptide Boc-Pro-Gly-Lys(*t*Bu)-Ala-OH was coupled with β-lactone TFA salt **2** to afford the desired pentapeptide following the general procedure C. Purification of the crude product using preparative HPLC (10 to 50% solvent B over 20 min, Higgins Analytical Proto 200 5 µm 250 × 10 nm C18 column) afforded peptide **13** as a white solid after lyophilization (7.2 mg, 32%).

HPLC (Higgins Analytical Proto 200 5 µm 150 × 2.0 nm C18 column, water/acetonitrile = 95/05 to 70/30 over 20 min, flow rate = 0.5 mL/min, λ = 214 nm), tR= 5.12 min.

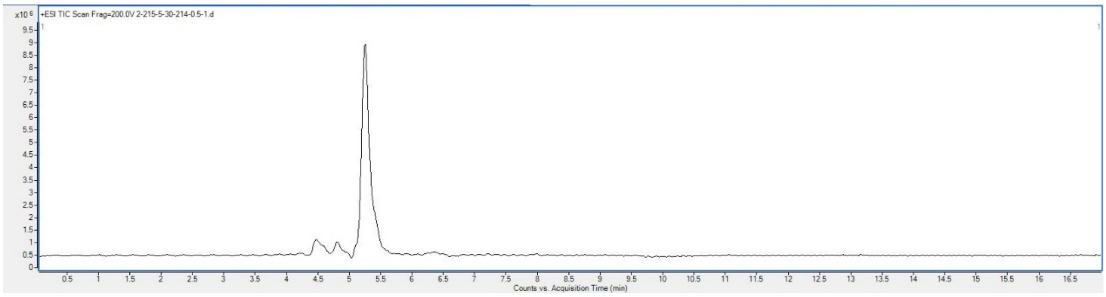

HRMS (ESI) m/z: $[M+H]^+$ Calcd for $C_{20}H_{34}N_6O_6$ 455.2613; Found 455.2664.

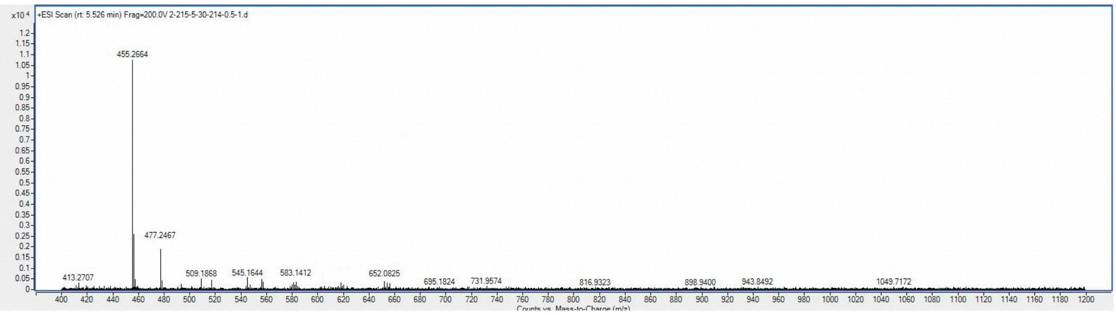

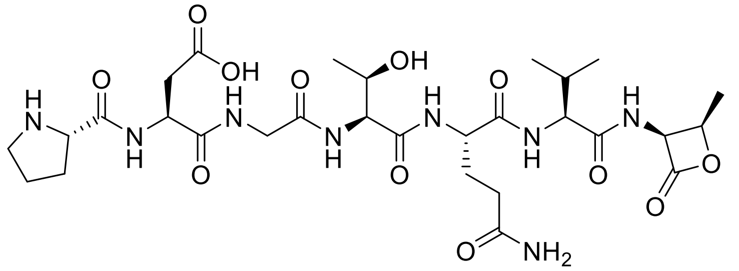

Chemical Formula: $C_{29}H_{46}N_8O_{12}$
Exact Mass: 698.3235
16

According to the general procedure A and B, the hexapeptide Boc-Pro-Asp(*t*Bu)-Gly-Thr(*t*Bu)-Gln(Trt)-Val-OH was generated directly from SPPS on a 0.08 mmol scale. The resulting hexapeptide Boc-Pro-Asp(*t*Bu)-Gly-Thr(*t*Bu)-Gln(Trt)-Val-OH was coupled with *β*-lactone TFA salt **2** to afford the desired polypeptide following the general procedure C. Purification of the crude product using preparative HPLC (10 to 50% solvent B over 20 min, Higgins Analytical Proto 200 5 µm 250 × 10 nm C18 column) afforded peptide **16** as a white solid after lyophilization (8.4 mg, 44%).

HPLC (Higgins Analytical Proto 200 5 µm 150 × 2.0 nm C18 column, water/acetonitrile = 90/10 to 40/60 over 20 min, flow rate = 0.5 mL/min, λ = 214 nm), tR= 11.18 min.

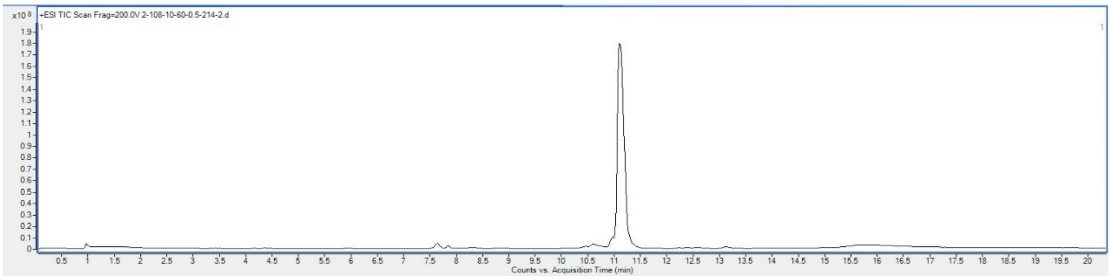

HRMS (ESI) m/z: $[M+H]^+$ Calcd for $C_{29}H_{46}N_8O_{12}$ 699.3308; Found 699.3383.

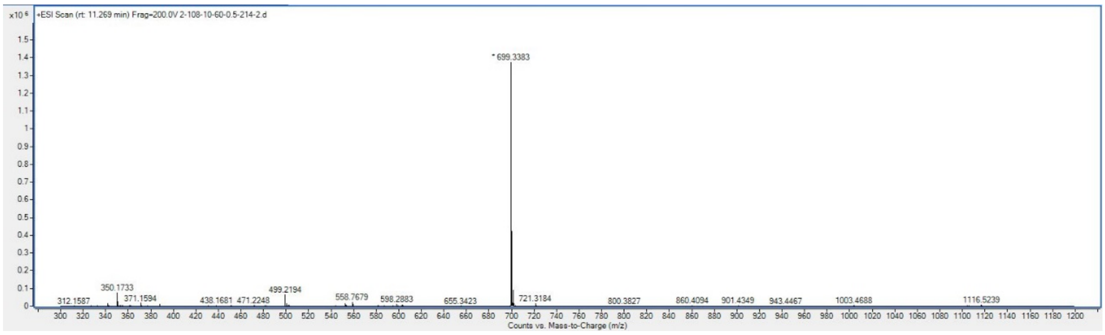

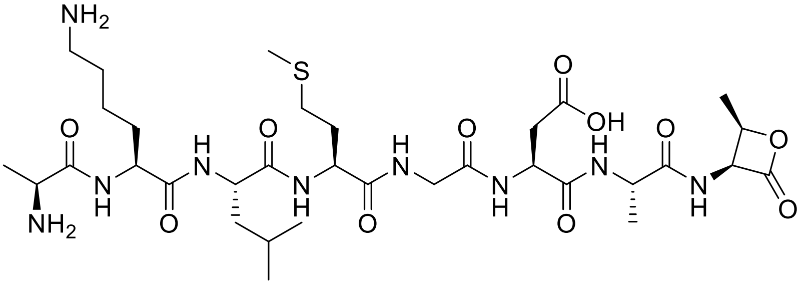

Chemical Formula: $C_{33}H_{57}N_9O_{11}S$
Exact Mass: 787.3898
22

According to the general procedure A and B, the heptapeptide Boc-Ala-Lys(*t*Bu)-Leu-Met-Gly-Asp(*t*Bu)-Ala-OH was generated directly from SPPS on a 0.08 mmol scale. The resulting heptapeptide Boc-Ala-Lys(*t*Bu)-Leu-Met-Gly-Asp(*t*Bu)-Ala-OH was coupled with β-lactone TFA salt **2** to afford the desired polypeptide following the general procedure C. Purification of the crude product using preparative HPLC (10 to 50% solvent B over 20 min, Higgins Analytical Proto 200 5 μm 250 × 10 nm C18 column) afforded peptide **22** as a white solid after lyophilization (10.1 mg, 38%).

HPLC (Higgins Analytical Proto 200 5 μm 150 × 2.0 nm C18 column, water/acetonitrile = 90/10 to 40/60 over 20 min, flow rate = 0.5 mL/min, λ = 214 nm), tR= 10.66 min.

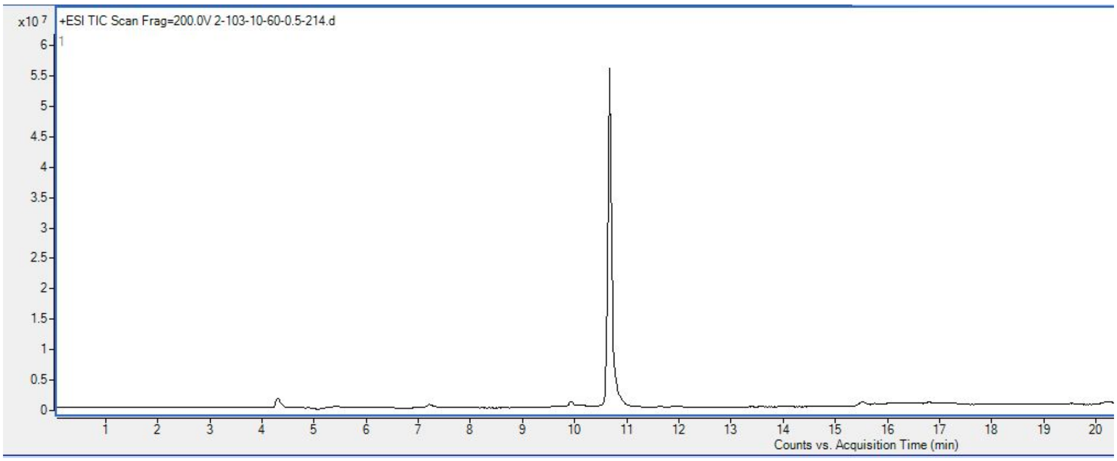

HRMS (ESI) m/z: $[M+H]^+$ Calcd for $C_{33}H_{57}N_9O_{11}S$ 788.3971; Found 788.4055.

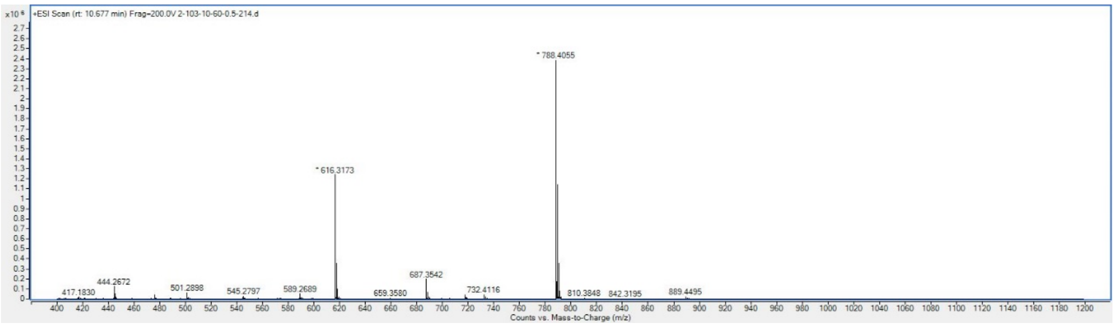

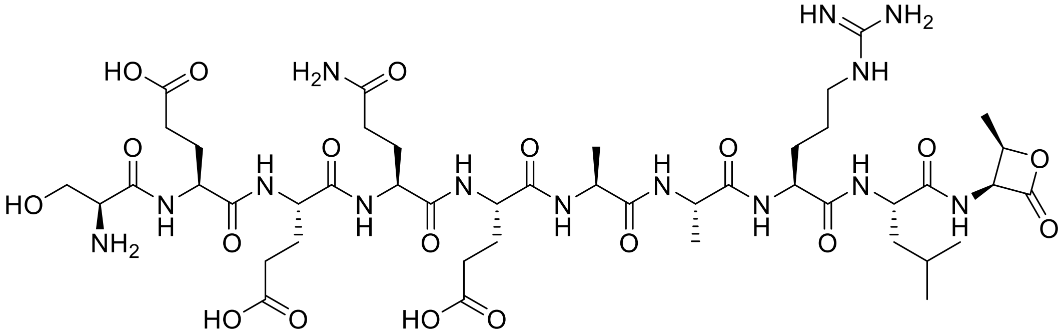

Chemical Formula: $C_{45}H_{74}N_{14}O_{19}$
Exact Mass: 1114.5255
26

According to the general procedure A and B, the nonapeptide Boc-Ser(*t*Bu)-Glu(*t*Bu)-Glu(*t*Bu)-Gln(Trt)-Glu(*t*Bu)-Ala-Ala-Arg(Pbf)-Leu-OH was generated directly from SPPS on a 0.08 mmol scale. The resulting nonapeptide Boc-Ser(*t*Bu)-Glu(*t*Bu)-Glu(*t*Bu)-Gln(Trt)-Glu(*t*Bu)-Ala-Ala-Arg(Pbf)-Leu-OH was coupled with *β*-lactone TFA salt **2** to afford the desired polypeptide following the general procedure C. Purification of the crude product using preparative HPLC (10 to 60% solvent B over 20 min, Higgins Analytical Proto 200 5 μm 250 × 10 nm C18 column) afforded peptide **26** as a white solid after lyophilization (7.1 mg, 27%).

HPLC (Higgins Analytical Proto 200 5 μm 150 × 2.0 nm C18 column, water/acetonitrile = 80/20 to 30/70 over 20 min, flow rate = 0.5 mL/min, λ = 214 nm), tR= 12.34 min.

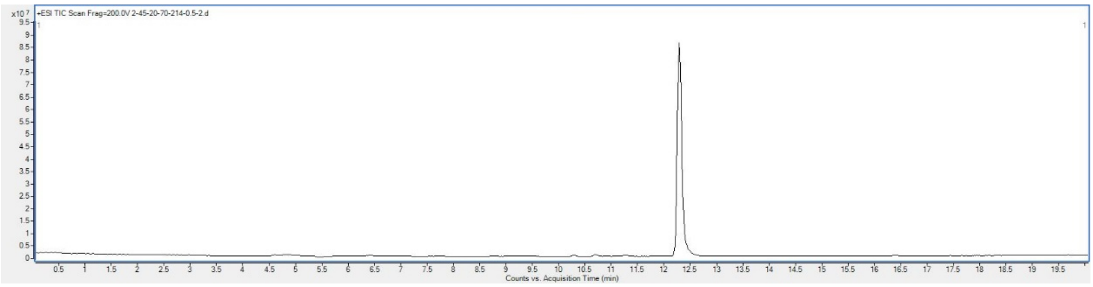

HRMS (ESI) m/z: $[M+H]^+$ Calcd for $C_{45}H_{74}N_{14}O_{19}$ 1115.5327; Found 1115.5485.

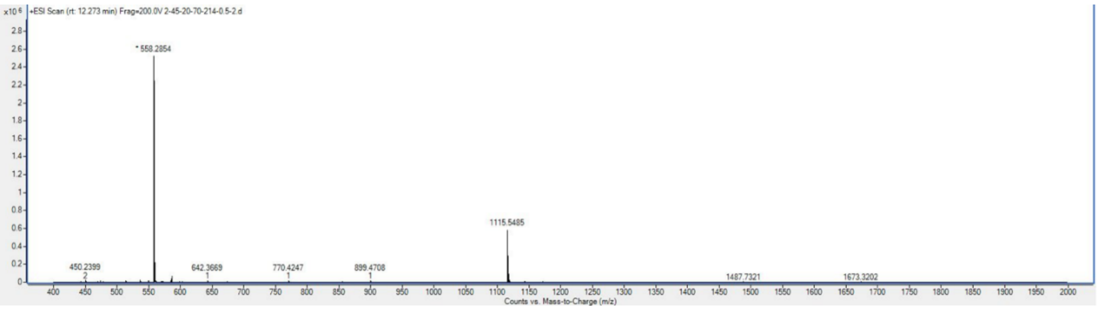

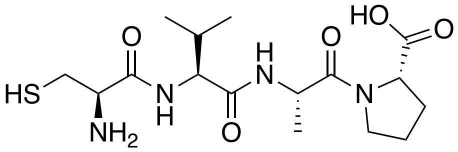

Chemical Formula: $C_{16}H_{28}N_4O_5S$
Exact Mass: 388.1780
4

According to the general procedure A and B, the tetrapeptide **4** was generated directly from SPPS on a 0.04 mmol scale by Biotage peptide synthesis system. Deprotection by TFA/TIPS/H_2O (95:2.5:2.5, *v*:*v*:*v*). Purification of the crude product using preparative HPLC (10 to 30% solvent B over 20 min, Higgins Analytical Proto 200 5 μm 250 × 10 nm C18 column) afforded peptide **4** as a white solid after lyophilization (10.1 mg, 75%).

HPLC (Higgins Analytical Proto 200 5 μm 150 × 2.0 nm C18 column, water/acetonitrile = 80/20 to 30/70 over 20 min, flow rate = 0.5 mL/min, λ = 214 nm), tR= 6.52 min.

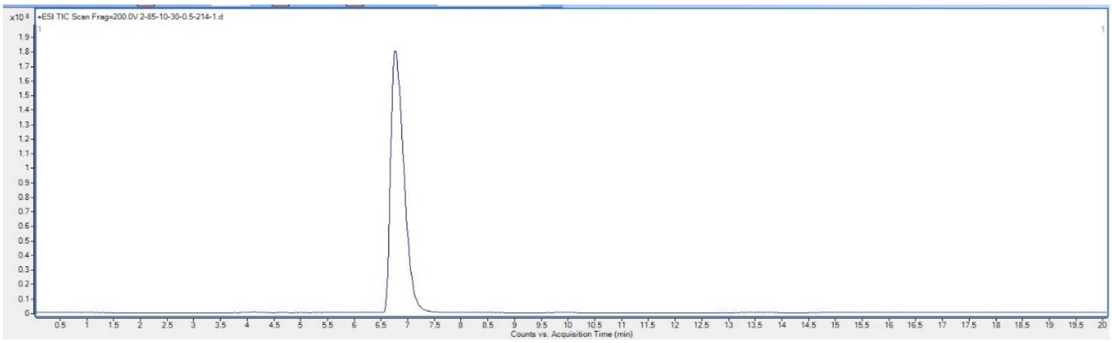

HRMS (ESI) m/z: $[M+H]^+$ Calcd for $C_{16}H_{28}N_4O_5S$ 389.1853; Found 389.2090.

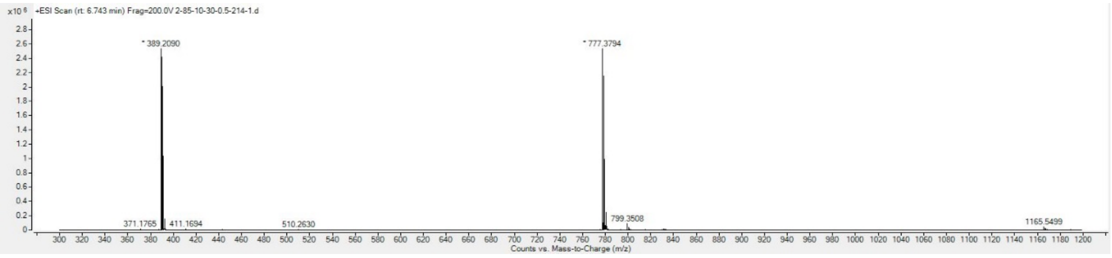

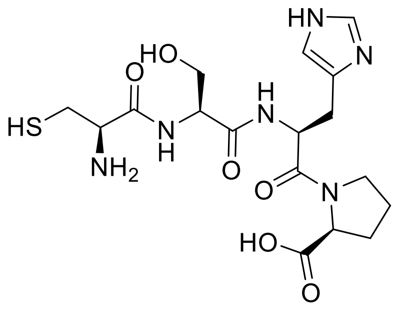

Chemical Formula: $C_{17}H_{26}N_6O_6S$
Exact Mass: 442.1635
9

According to the general procedure A and B, the tetrapeptide **9** was generated directly from SPPS on a 0.04 mmol scale by Biotage peptide synthesis system. Deprotection by TFA/TIPS/H_2O (95:2.5:2.5, *v*:*v*:*v*). Purification of the crude product using preparative HPLC (10 to 50% solvent B over 20 min, Higgins Analytical Proto 200 5 μm 250 × 10 nm C18 column) afforded peptide **9** as a white solid after lyophilization (11.7 mg, 66%).

HPLC (Higgins Analytical Proto 200 5 μm 150 × 2.0 nm C18 column, water/acetonitrile = 80/20 to 40/60 over 20 min, flow rate = 0.5 mL/min, λ = 214 nm), tR= 13.46 min.

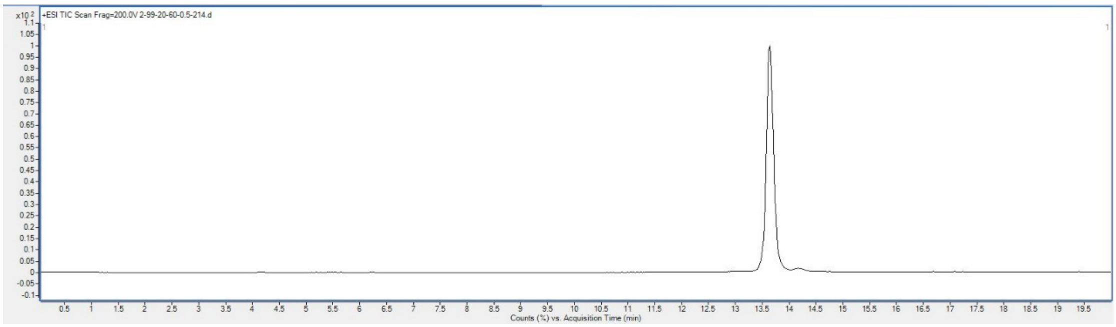

HRMS (ESI) m/z: $[M+H]^+$ Calcd for $C_{17}H_{26}N_6O_6S$ 443.1707; Found 443.1737.

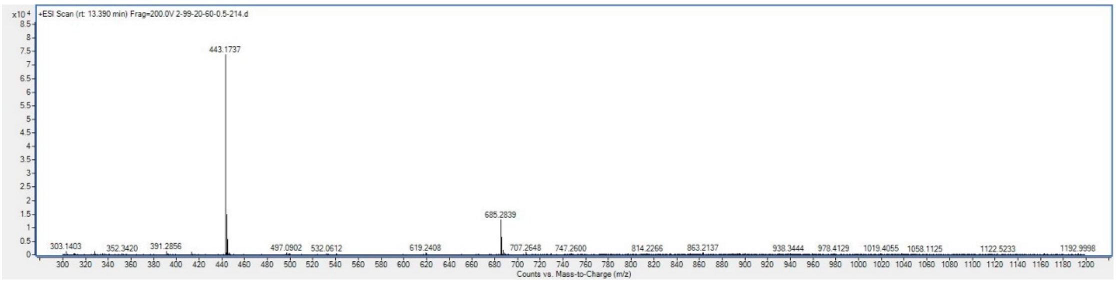

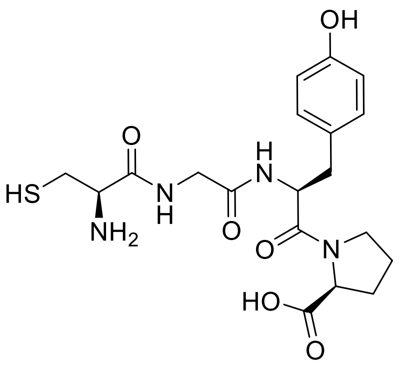

Chemical Formula: $C_{19}H_{26}N_4O_6S$
Exact Mass: 438.1573
11

According to the general procedure A and B, the tetrapeptide **11** was generated directly from SPPS on a 0.04 mmol scale by Biotage peptide synthesis system. Deprotection by TFA/TIPS/H_2O (95:2.5:2.5, *v*:*v*:*v*). Purification of the crude product using preparative HPLC (10 to 50% solvent B over 20 min, Higgins Analytical Proto 200 5 µm 250 × 10 nm C18 column) afforded peptide **11** as a white solid after lyophilization (12.4 mg, 71%).

HPLC (Higgins Analytical Proto 200 5 µm 150 × 2.0 nm C18 column, water/acetonitrile = 90/10 to 60/40 over 20 min, flow rate = 0.5 mL/min, λ = 214 nm), tR= 11.39 min.

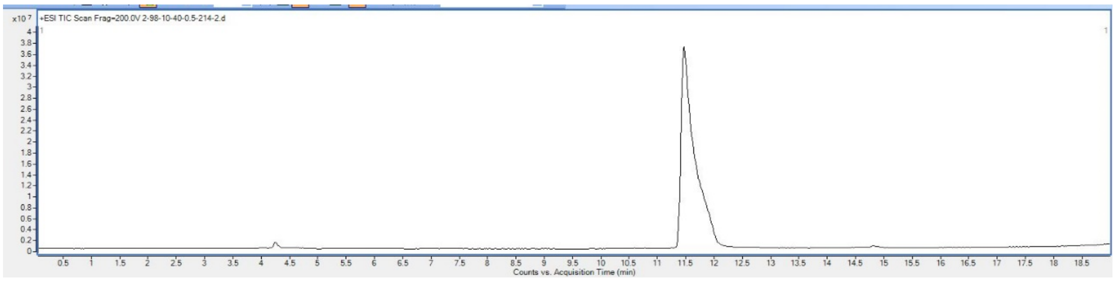

HRMS (ESI) m/z: $[M+H]^+$ Calcd for $C_{19}H_{26}N_4O_6S$ 439.1646; Found 439.1716.

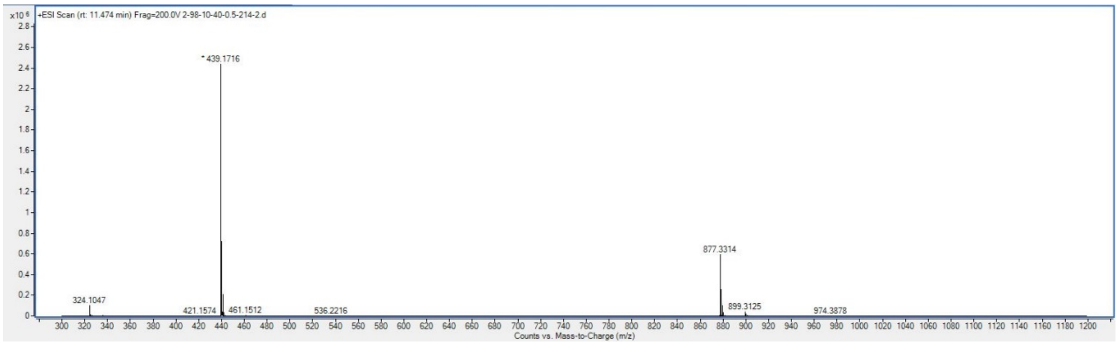

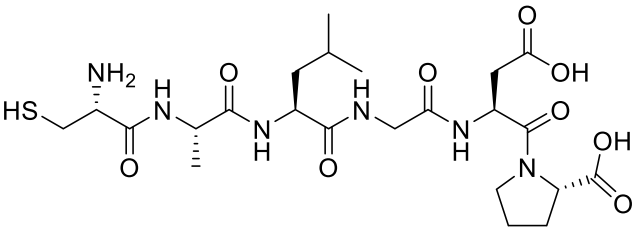

Chemical Formula: $C_{23}H_{38}N_6O_9S$
Exact Mass: 574.2421
14

According to the general procedure A and B, the hexapeptide **14** was generated directly from SPPS on a 0.04 mmol scale by Biotage peptide synthesis system. Deprotection by TFA/TIPS/H_2O (95:2.5:2.5, *v*:*v*:*v*). Purification of the crude product using preparative HPLC (10 to 50% solvent B over 20 min, Higgins Analytical Proto 200 5 μm 250 × 10 nm C18 column) afforded peptide **14** as a white solid after lyophilization (17.3 mg, 75%).

HPLC (Higgins Analytical Proto 200 5 μm 150 × 2.0 nm C18 column, water/acetonitrile = 90/10 to 30/70 over 20 min, flow rate = 0.5 mL/min, λ = 214 nm), tR= 11.62 min.

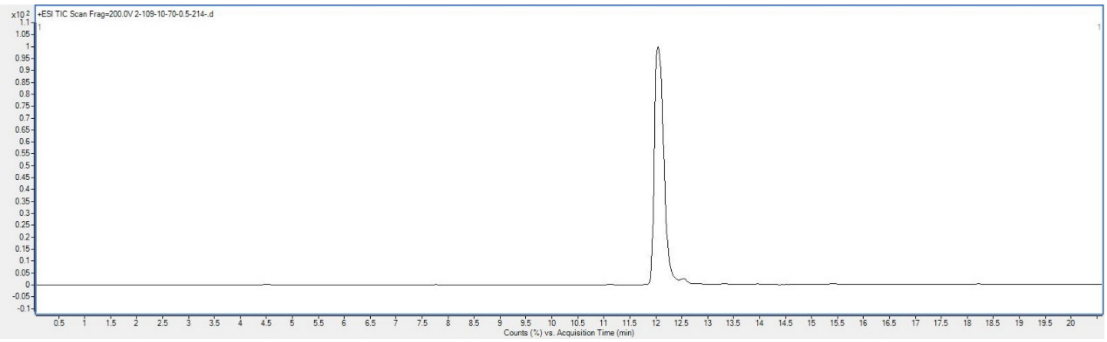

HRMS (ESI) m/z: $[M+H]^+$ Calcd for $C_{23}H_{38}N_6O_9S$ 575.2494; Found 575.2562.

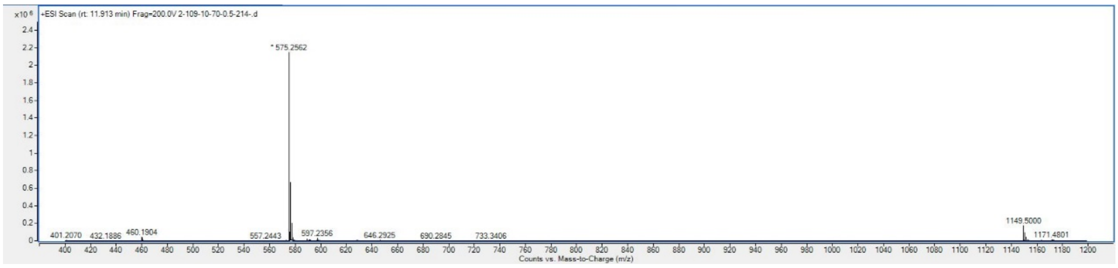

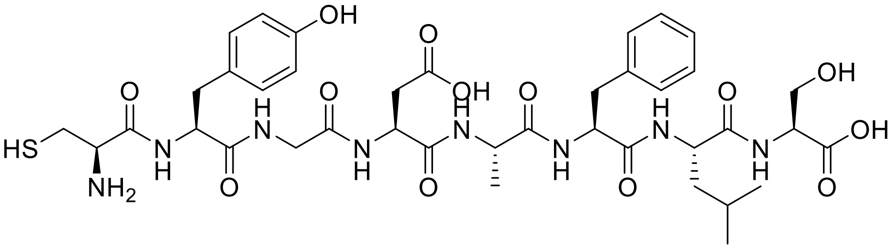

Chemical Formula: $C_{39}H_{54}N_8O_{13}S$
Exact Mass: 874.3531
17

According to the general procedure A and B, the octapeptide **17** was generated directly from SPPS on a 0.04 mmol scale by Biotage peptide synthesis system. Deprotection by TFA/TIPS/H_2O (95:2.5:2.5, *v*:*v*:*v*). Purification of the crude product using preparative HPLC (10 to 50% solvent B over 20 min, Higgins Analytical Proto 200 5 µm 250 × 10 nm C18 column) afforded peptide **17** as a white solid after lyophilization (22.7 mg, 65%).

HPLC (Higgins Analytical Proto 200 5 µm 150 × 2.0 nm C18 column, water/acetonitrile = 80/20 to 40/60 over 20 min, flow rate = 0.5 mL/min, λ = 214 nm), tR= 11.56 min.

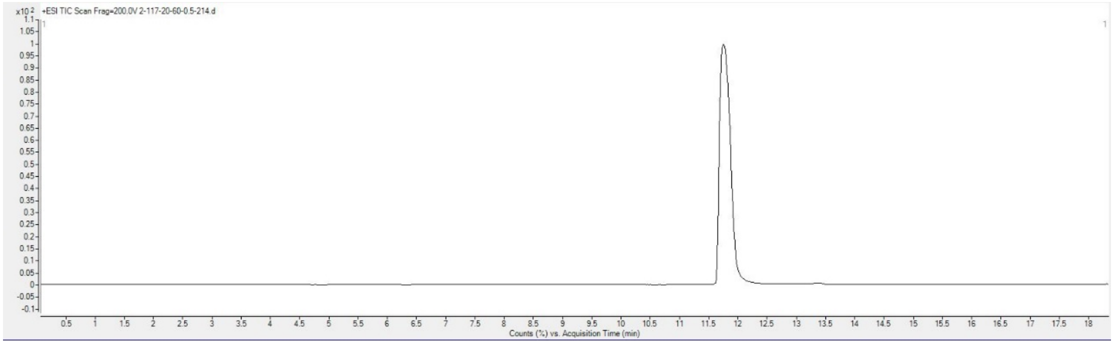

HRMS (ESI) m/z: $[M+H]^+$ Calcd for $C_{39}H_{54}N_8O_{13}S$ 875.3604; Found 875.3737.

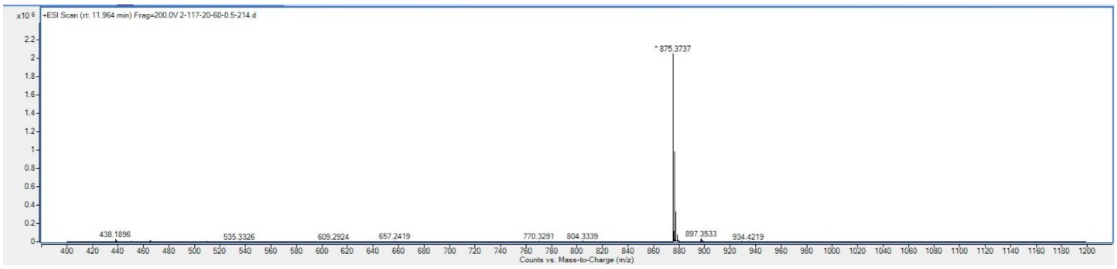

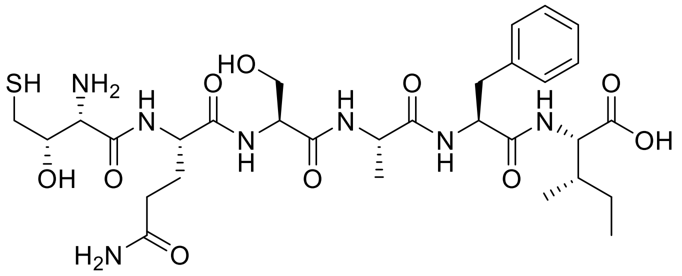

Chemical Formula: $C_{30}H_{47}N_7O_{10}S$
Exact Mass: 697.3105
20

According to the general procedure A and B, the hexapeptide **20** was generated directly from SPPS on a 0.04 mmol scale by Biotage peptide synthesis system. Deprotection by TFA/TIPS/H_2O (95:2.5:2.5, *v*:*v*:*v*). Purification of the crude product using preparative HPLC (10 to 60% solvent B over 20 min, Higgins Analytical Proto 200 5 μm 250 × 10 nm C18 column) afforded peptide **20** as a white solid after lyophilization (18.0 mg, 54%).

HPLC (Higgins Analytical Proto 200 5 μm 150 × 2.0 nm C18 column, water/acetonitrile = 80/20 to 40/60 over 20 min, flow rate = 0.5 mL/min, λ = 214 nm), tR= 11.37 min.

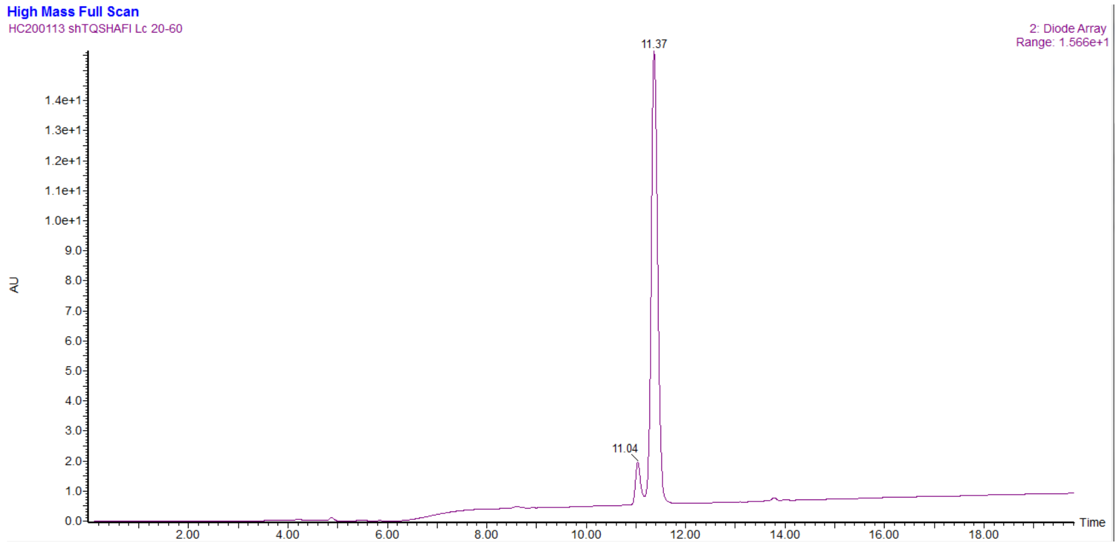

MS (ESI) m/z: $[M+H]^+$ Calcd for $C_{30}H_{47}N_7O_{10}S$ 698.3178; Found 698.5526.

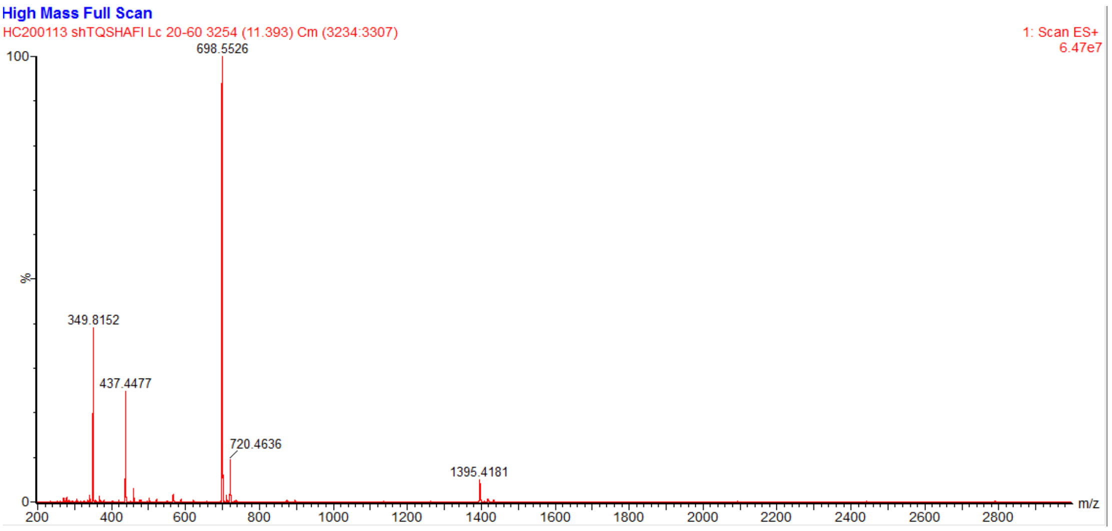

ESI-MS of impurity at tR= 11.04 min. This is not the epimer of **20**.

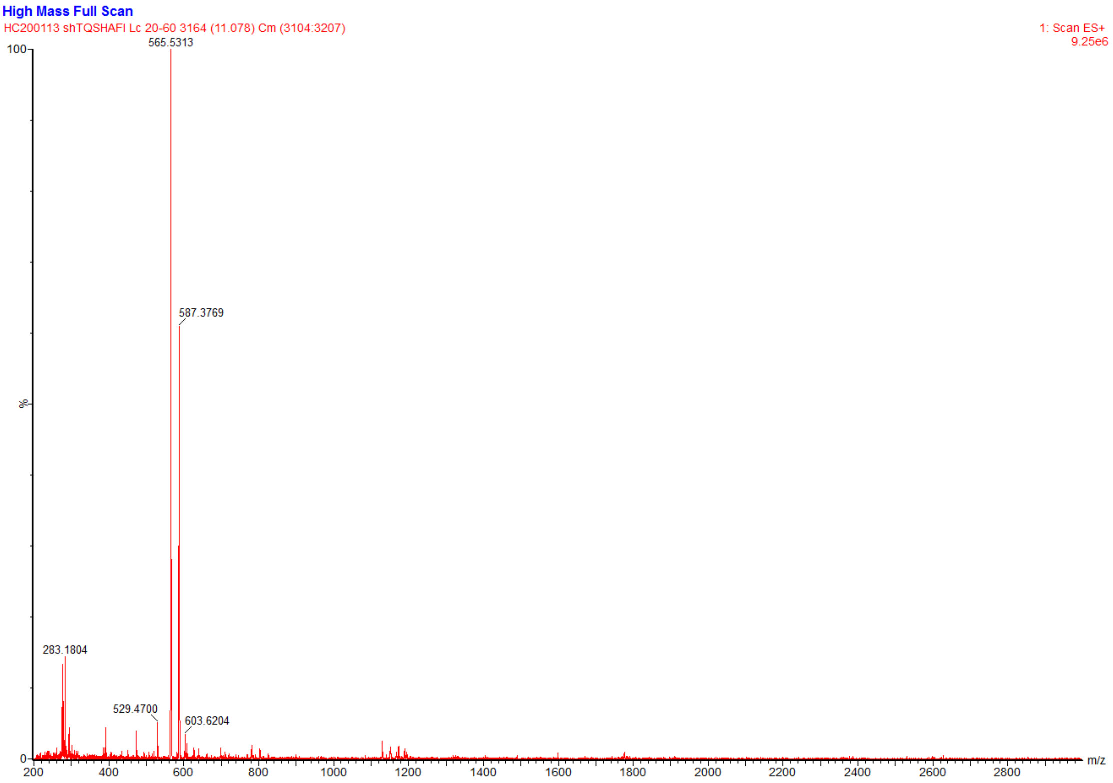

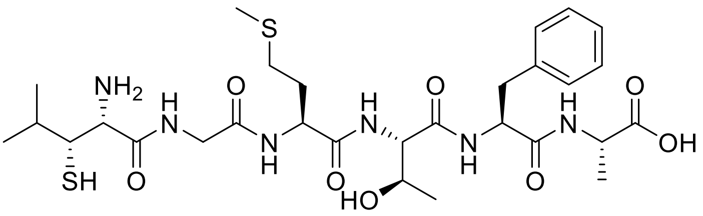

Chemical Formula: $C_{29}H_{46}N_6O_8S_2$
Exact Mass: 670.2819
24

According to the general procedure A and B, the hexapeptide **24** was generated directly from SPPS on a 0.04 mmol scale by Biotage peptide synthesis system. Deprotection by

TFA/TIPS/H_2O (95:2.5:2.5, *v*:*v*:*v*). Purification of the crude product using preparative HPLC (10 to 50% solvent B over 20 min, Higgins Analytical Proto 200 5 µm 250 × 10 nm C18 column) afforded peptide **24** as a white solid after lyophilization (16.5 mg, 62%).

HPLC (Higgins Analytical Proto 200 5 µm 150 × 2.0 nm C18 column, water/acetonitrile = 90/10 to 40/60 over 20 min, flow rate = 0.5 mL/min, λ = 214 nm), tR= 10.35 min.

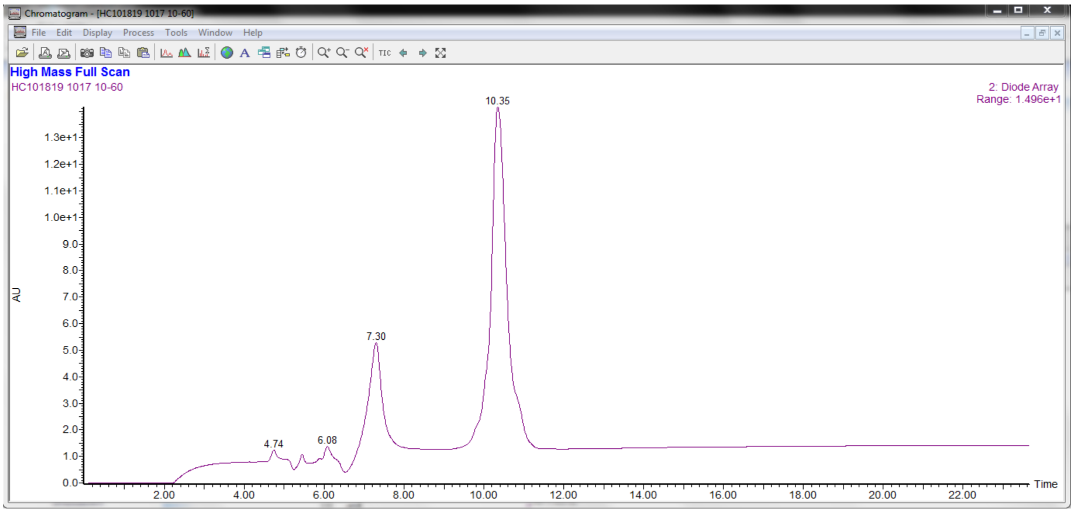

MS (ESI) m/z: $[M+H]^+$ Calcd for $C_{29}H_{46}N_6O_8S_2$ 671.2891; Found 670.9166.

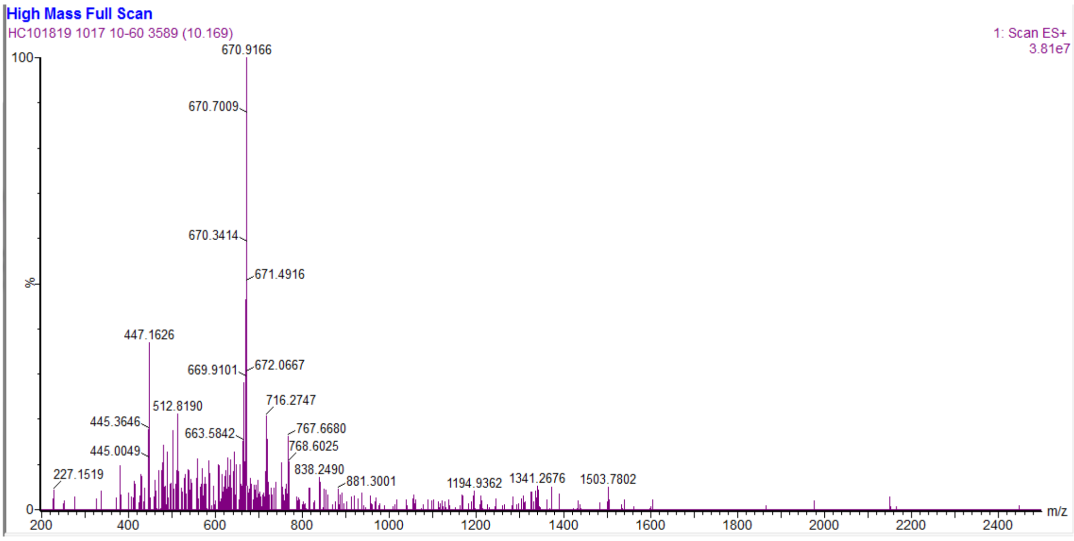

ESI-MS of impurity at tR= 7.30 min. This is the injection peak.

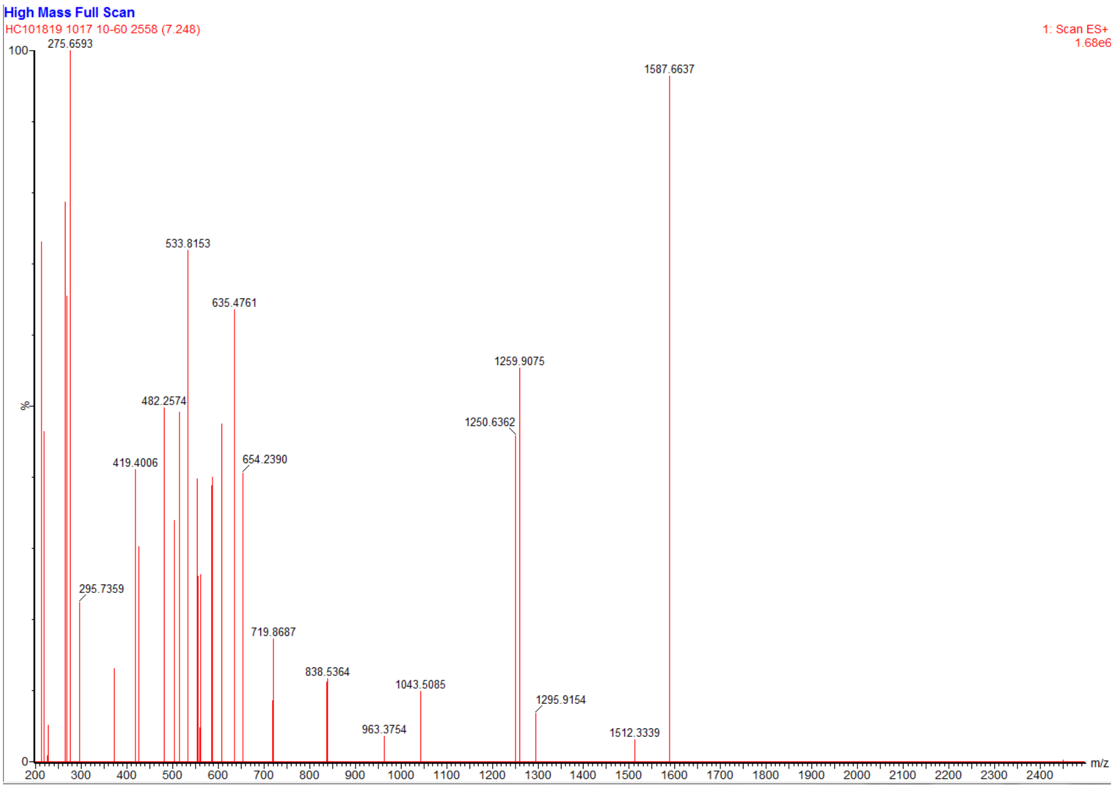

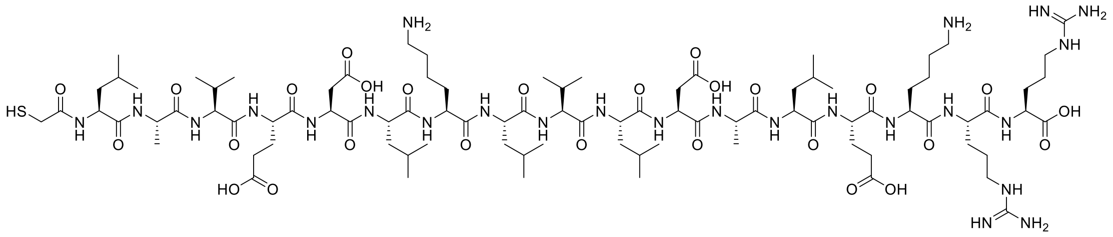

Chemical Formula: $C_{90}H_{159}N_{25}O_{27}S$
Exact Mass: 2054.1558
27

According to the general procedure A and B, the polypeptide **27** was generated directly from SPPS on a 0.04 mmol scale by Biotage peptide synthesis system. Deprotection by TFA/TIPS/H_2O (95:2.5:2.5, *v*:*v*:*v*). Purification of the crude product using preparative HPLC (10 to 50% solvent B over 20 min, Higgins Analytical Proto 200 5 µm 250 × 10 nm C18 column) afforded peptide **27** as a white solid after lyophilization (37.0 mg, 45%).

HPLC (Higgins Analytical Proto 200 5 µm 150 × 2.0 nm C18 column, water/acetonitrile = 80/20 to 30/70 over 20 min, flow rate = 0.5 mL/min, λ = 214 nm), tR= 16.48 min.

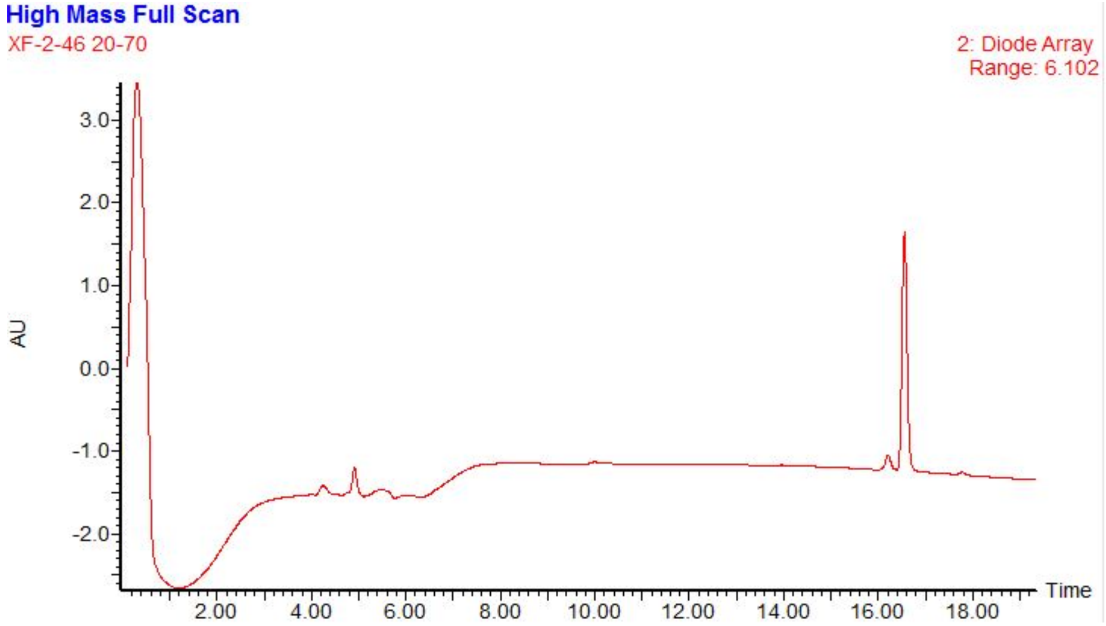

MS (ESI) m/z: $[M+H]^+$ Calcd for $C_{90}H_{159}N_{25}O_{27}S$ 2055.1631; Found 2055.3215.

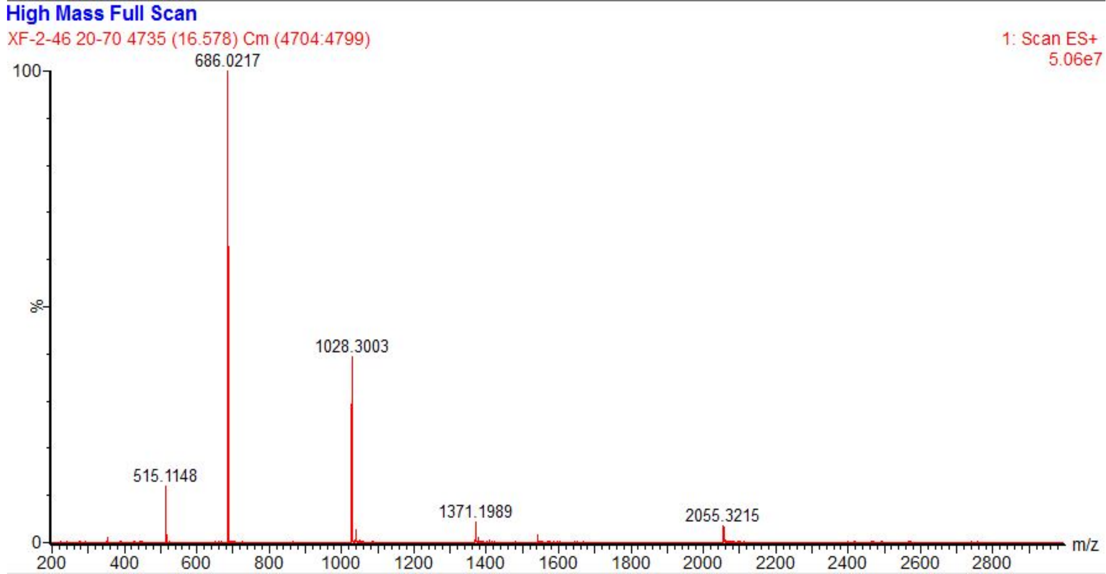

ESI-MS of impurity at tR= 16.20 min. This is not the epimer of **27**.

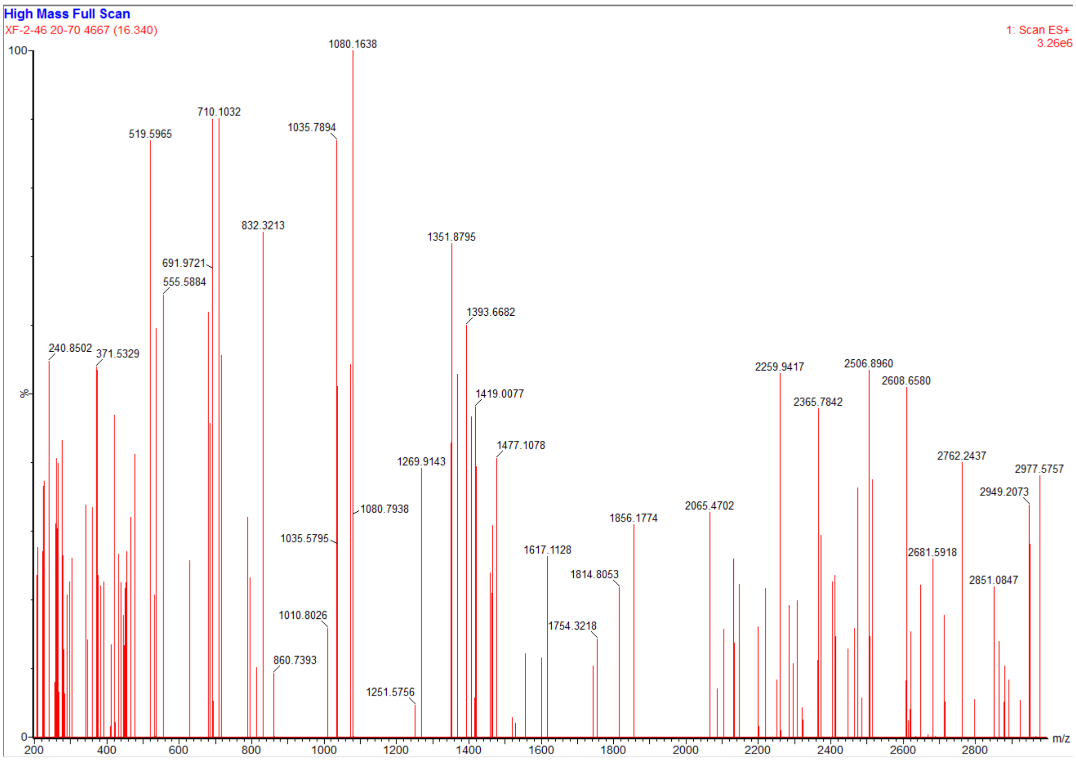

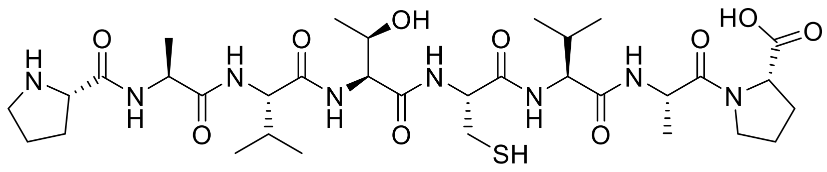

Chemical Formula: $C_{33}H_{56}N_8O_{10}S$
Exact Mass: 756.3840
5

Peptide **5** was prepared according to General Procedure D. 0.72 mg **3** (1.5 equiv.) and 0.50 mg **4** (1.0 equiv.) was dissolved in 200 µL Et_3N (pH = 10) solution. The resulting mixture was stirred for 1 h. Then, purification of the crude peptide by preparative HPLC (20% to 60% solvent B over 20min, Higgins Analytical Proto 200 10 µm 250 × 20nm C18 column) afforded peptide **5** as a white solid after lyophilization (0.80 mg, 82%). LC traces from crude reaction mixtures:

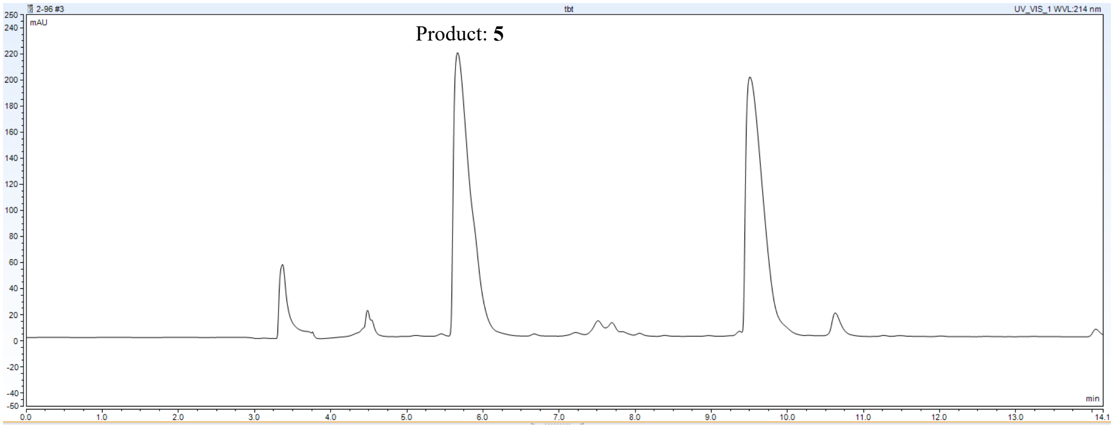

HPLC (Higgins Analytical Proto 200 5 µm 150 × 2.0 nm C18 column, water/acetonitrile = 85/15 to 60/40 over 20 min, flow rate = 0.5 mL/min, λ = 214 nm), tR= 10.52 min.

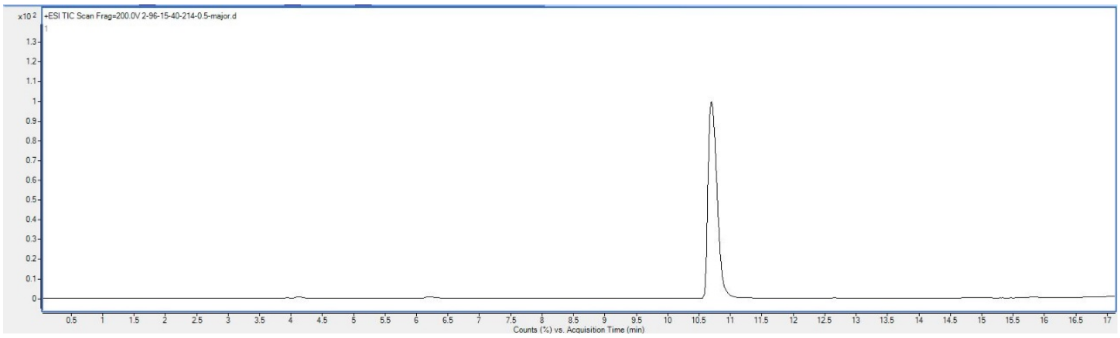

HRMS (ESI) m/z: $[M+H]^+$ Calcd for $C_{33}H_{56}N_8O_{10}S$ 757.3913; Found 757.4022.

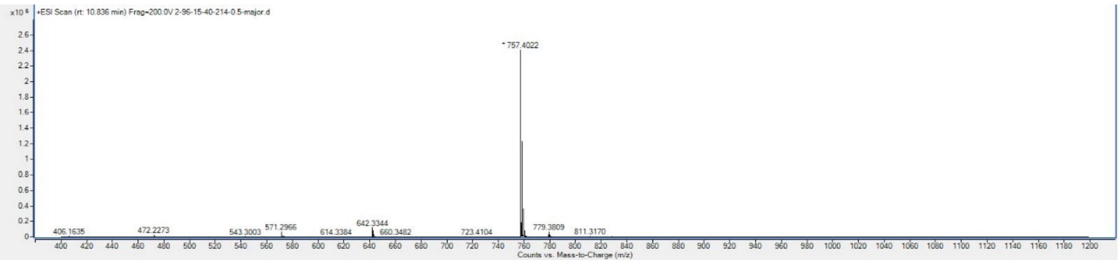

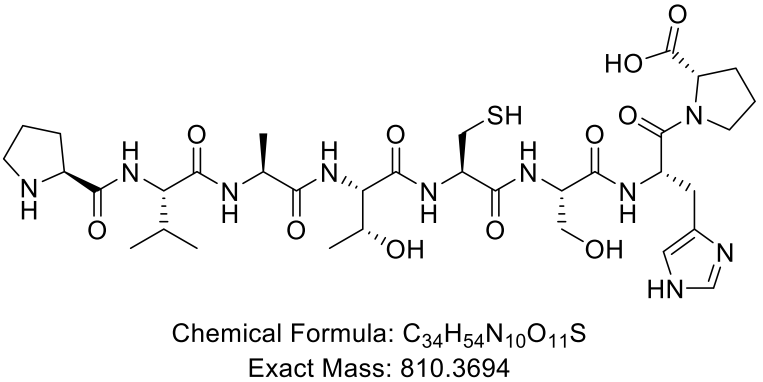

Chemical Formula: $C_{34}H_{54}N_{10}O_{11}S$

Exact Mass: 810.3694

10

Peptide **10** was prepared according to General Procedure D. 0.60 mg **3** (1.5 equiv.) and 0.51 mg **9** (1.0 equiv.) was dissolved in 200 µL Et_3N (pH = 10) solution. The resulting mixture was stirred for 0.5 h. Then, purification of the crude peptide by preparative

HPLC (20% to 60% solvent B over 20min, Higgins Analytical Proto 200 10 µm 250 × 20nm C18 column) afforded peptide **10** as a white solid after lyophilization (0.87 mg, 89%).

LC traces from crude reaction mixtures:

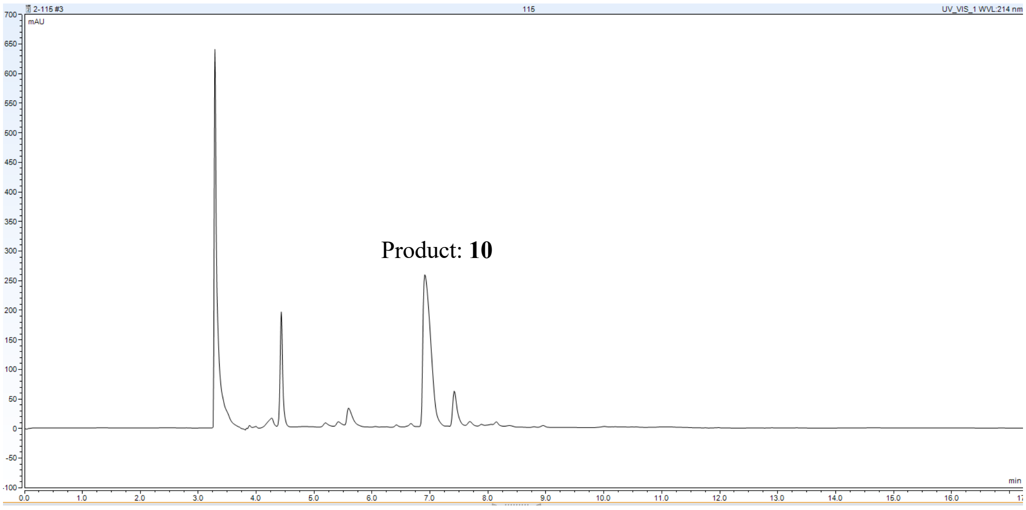

HPLC (Higgins Analytical Proto 200 5 µm 150 × 2.0 nm C18 column, water/acetonitrile = 90/10 to 30/70 over 20 min, flow rate = 0.5 mL/min, λ = 214 nm), tR= 10.39 min.

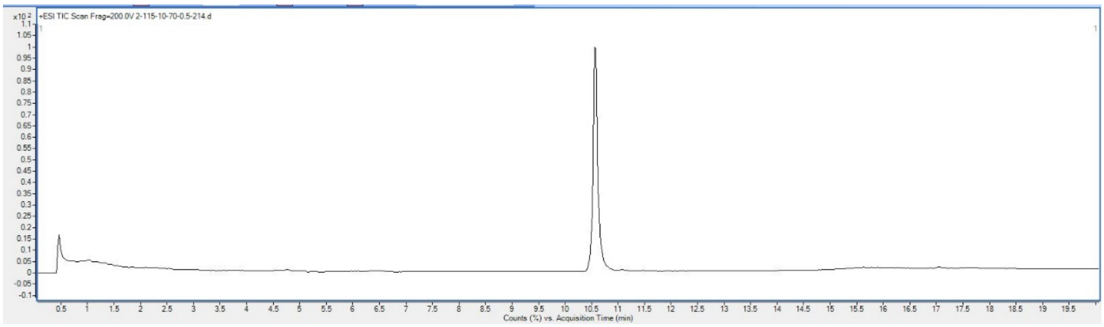

HRMS (ESI) m/z: $[M+H]^+$ Calcd for $C_{34}H_{54}N_{10}O_{11}S$ 811.3767; Found 811.3830.

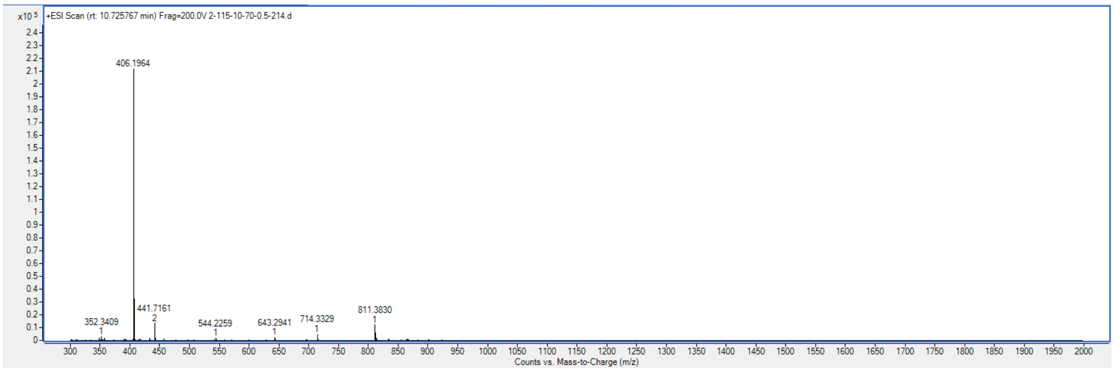

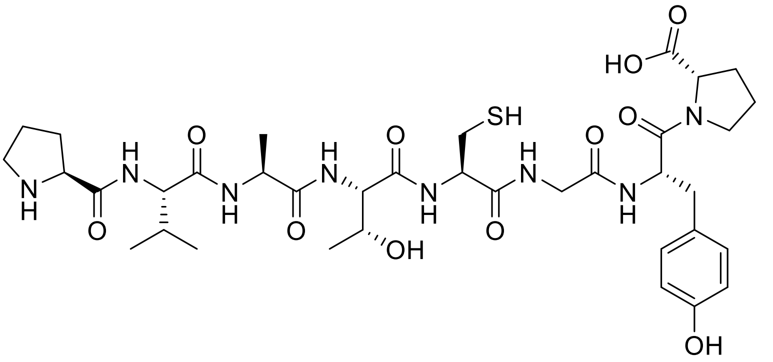

Chemical Formula: $C_{36}H_{54}N_8O_{11}S$
Exact Mass: 806.3633
12

Peptide **12** was prepared according to General Procedure D. 0.61 mg **3** (1.5 equiv.) and 0.51 mg **11** (1.0 equiv.) was dissolved in 200 µL Et_3N (pH = 10) solution. The resulting mixture was stirred for 1 h. Then, purification of the crude peptide by preparative HPLC (20% to 60% solvent B over 20min, Higgins Analytical Proto 200 10 µm 250 × 20nm C18 column) afforded peptide **12** as a white solid after lyophilization (0.87 mg, 93%).
LC traces from crude reaction mixtures:

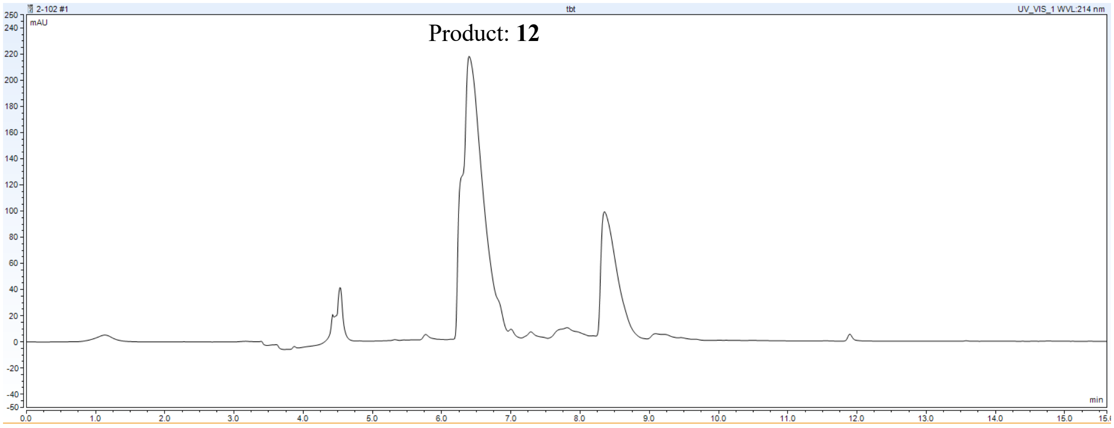

HPLC (Higgins Analytical Proto 200 5 µm 150 × 2.0 nm C18 column, water/acetonitrile = 85/15 to 40/60 over 20 min, flow rate = 0.5 mL/min, λ = 214 nm), tR= 10.88 min.

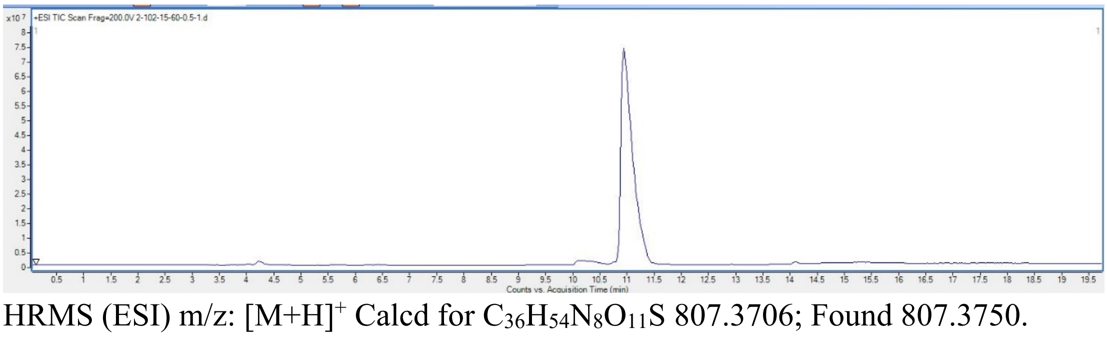

HRMS (ESI) m/z: $[M+H]^+$ Calcd for $C_{36}H_{54}N_8O_{11}S$ 807.3706; Found 807.3750.

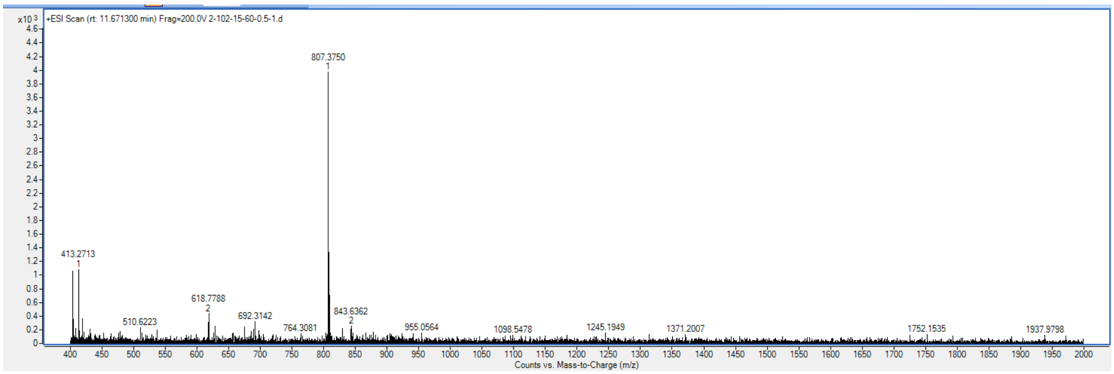

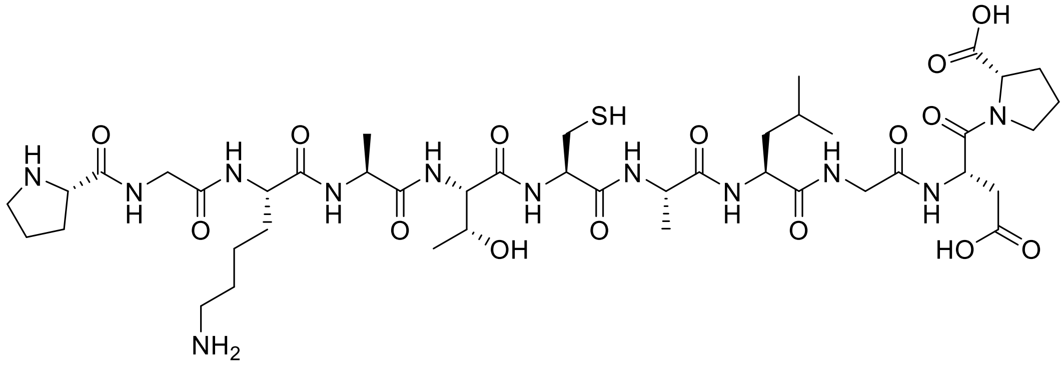

Chemical Formula: $C_{43}H_{72}N_{12}O_{15}S$

Exact Mass: 1028.4961

15

Peptide **15** was prepared according to General Procedure D. 0.61 mg **13** (1.5 equiv.) and 0.50 mg **14** (1.0 equiv.) was dissolved in 200 μL Et_3N (pH = 10) solution. The resulting mixture was stirred for 1 h. Then, purification of the crude peptide by preparative HPLC (20% to 60% solvent B over 20min, Higgins Analytical Proto 200 10 μm 250 × 20nm C18 column) afforded peptide **15** as a white solid after lyophilization (0.68 mg, 76%).

LC traces from crude reaction mixtures:

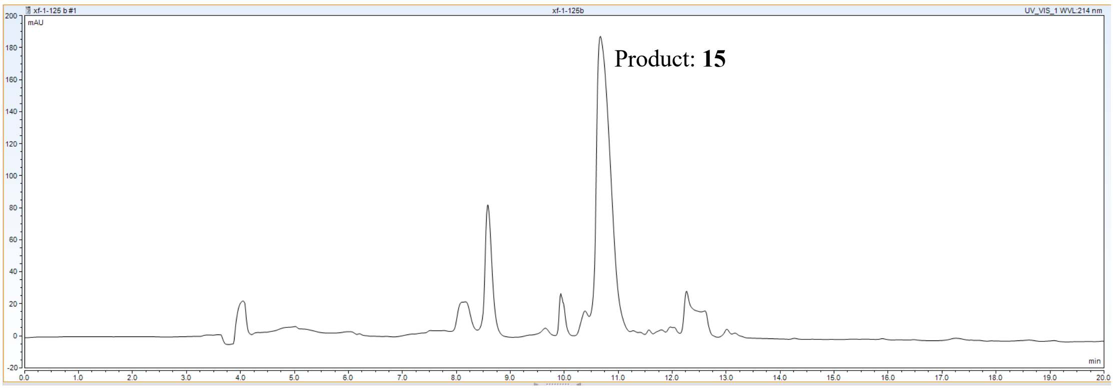

HPLC (Higgins Analytical Proto 200 5 μm 150 × 2.0 nm C18 column, water/acetonitrile = 90/10 to 50/50 over 20 min, flow rate = 0.5 mL/min, λ = 214 nm), tR= 12.50 min.

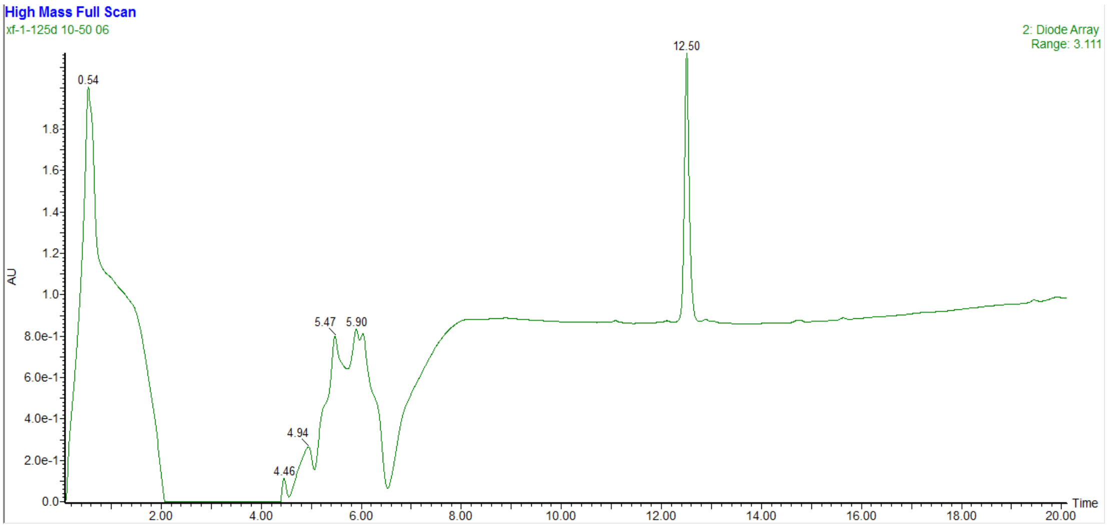

MS (ESI) m/z: [M+H]+ Calcd for $C_{43}H_{72}N_{12}O_{15}S$ 1029.5034; Found 1029.6379.

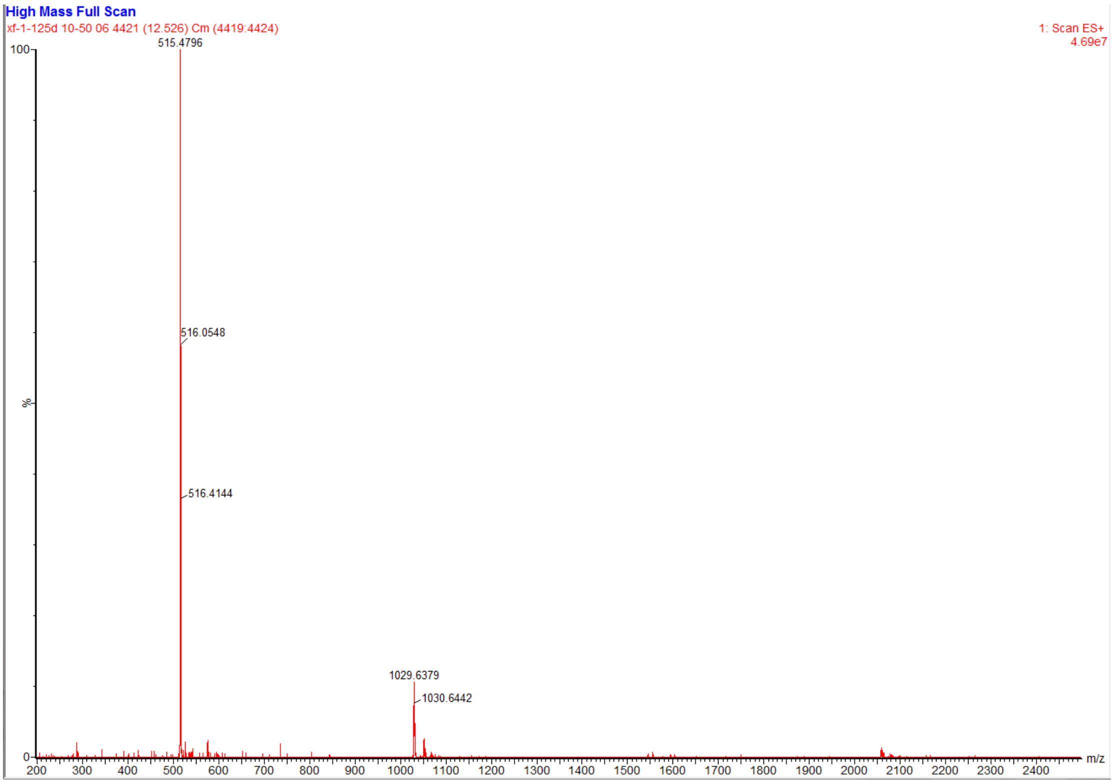

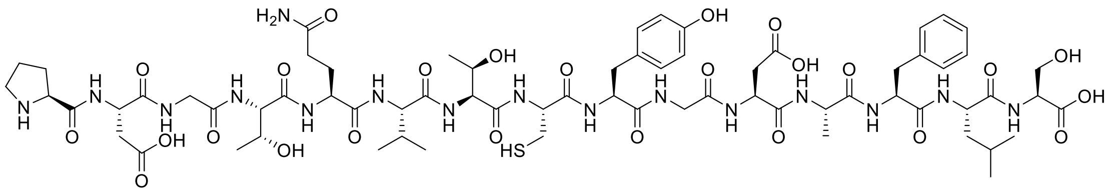

Chemical Formula: $C_{68}H_{100}N_{16}O_{25}S$
Exact Mass: 1572.6766
18

Peptide **18** was prepared according to General Procedure D. 0.60 mg **16** (1.5 equiv.) and 0.51 mg **17** (1.0 equiv.) was dissolved in 200 μL Et_3N (pH = 10) solution. The resulting mixture was stirred for 1 h. Then, purification of the crude peptide by preparative HPLC (20% to 60% solvent B over 20min, Higgins Analytical Proto 200

10 µm 250 × 20nm C18 column) afforded peptide **18** as a white solid after lyophilization (0.74 mg, 81%).

LC traces from crude reaction mixtures:

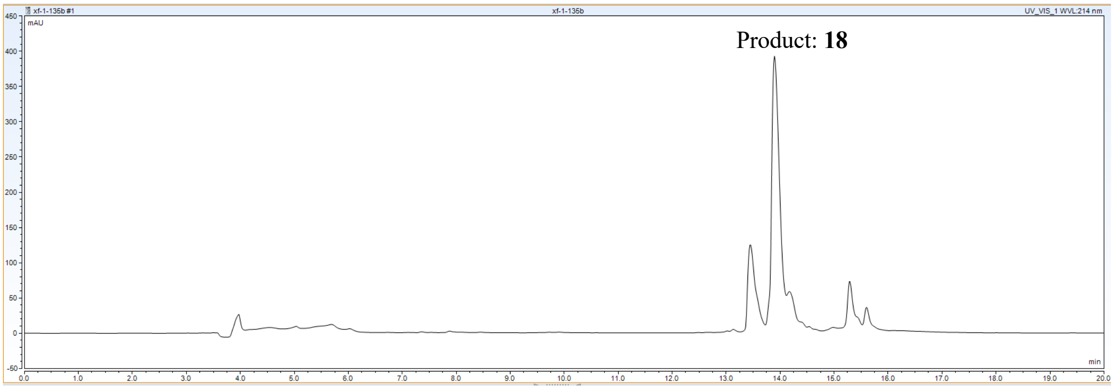

HPLC (Higgins Analytical Proto 200 5 µm 150 × 2.0 nm C18 column, water/acetonitrile = 80/20 to 50/50 over 20 min, flow rate = 0.5 mL/min, λ = 214 nm), tR= 13.20 min.

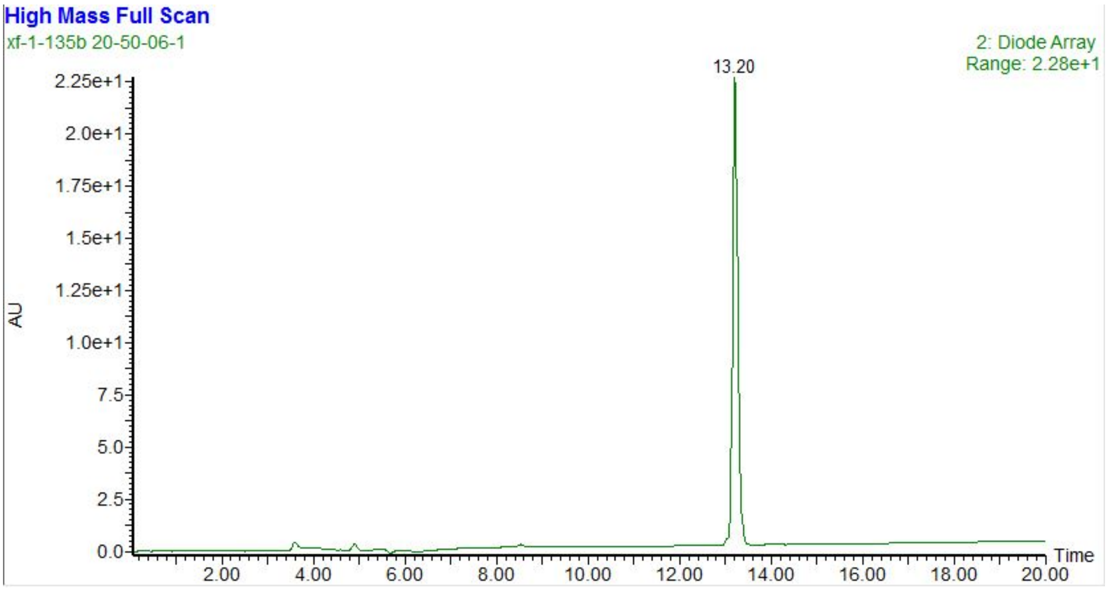

MS (ESI) m/z: $[M+H]^+$ Calcd for $C_{68}H_{100}N_{16}O_{25}S$ 1573.6839; Found 1573.5752.

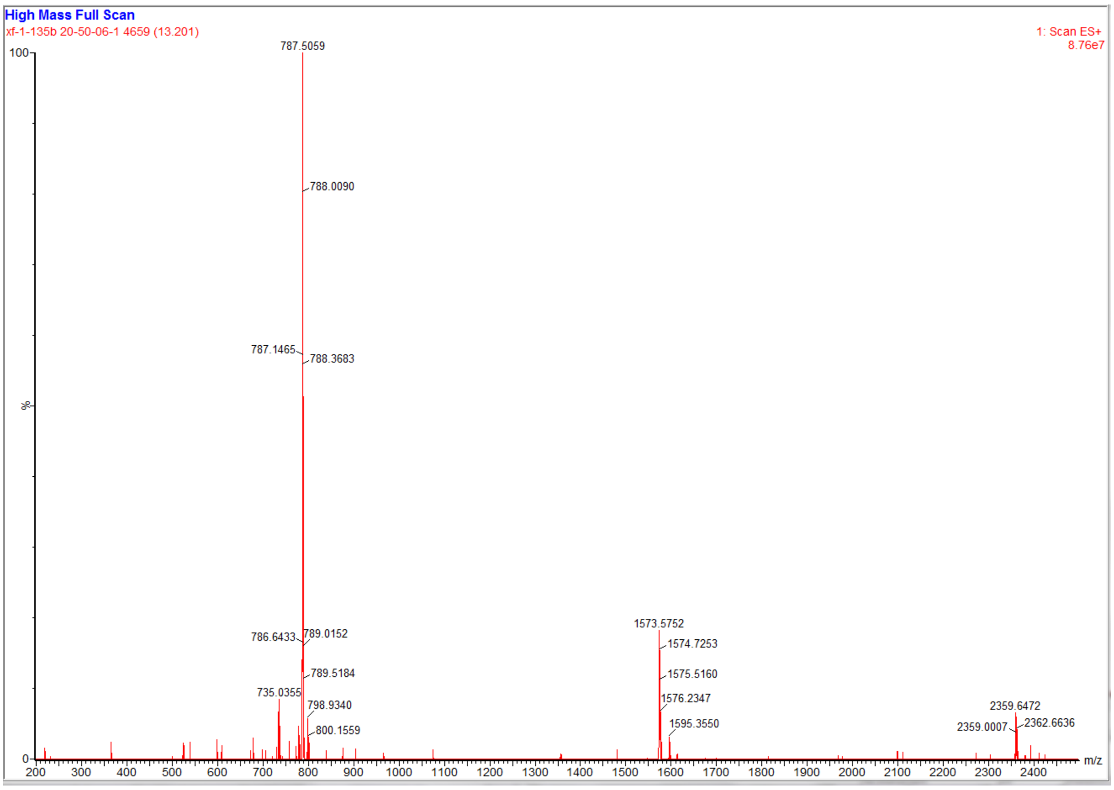

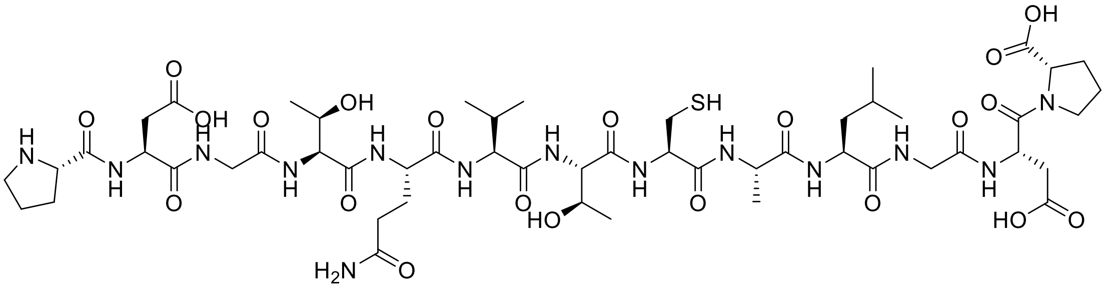

Chemical Formula: $C_{52}H_{84}N_{14}O_{21}S$
Exact Mass: 1272.5656
19

Peptide **19** was prepared according to General Procedure D. 0.89 mg **16** (1.5 equiv.) and 0.51 mg **14** (1.0 equiv.) was dissolved in 200 μL Et_3N (pH = 10) solution. The resulting mixture was stirred for 1 h. Then, purification of the crude peptide by preparative HPLC (20% to 60% solvent B over 20min, Higgins Analytical Proto 200 10 μm 250 × 20nm C18 column) afforded peptide **19** as a white solid after lyophilization (0.71 mg, 63%).

LC traces from crude reaction mixtures:

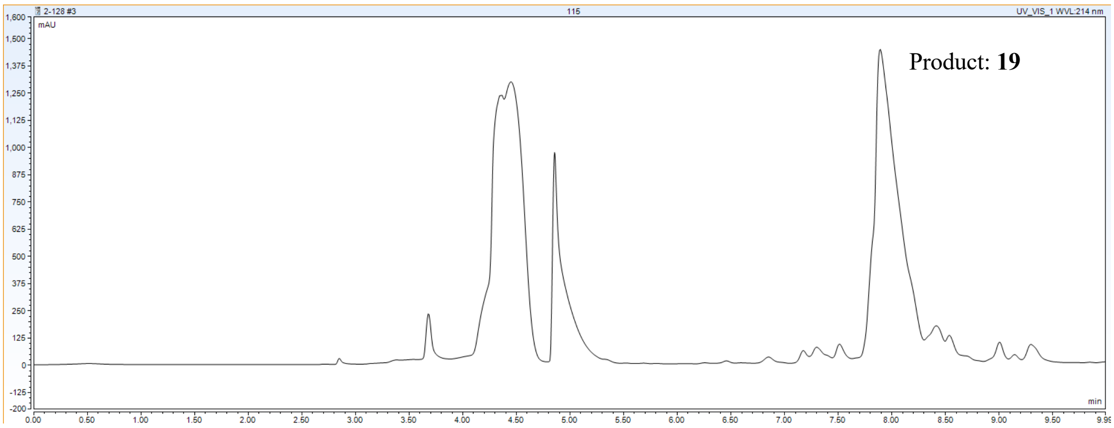

HPLC (Higgins Analytical Proto 200 5 μm 150 × 2.0 nm C18 column, water/acetonitrile = 80/20 to 40/60 over 20 min, flow rate = 0.5 mL/min, λ = 214 nm), tR= 11.52 min.

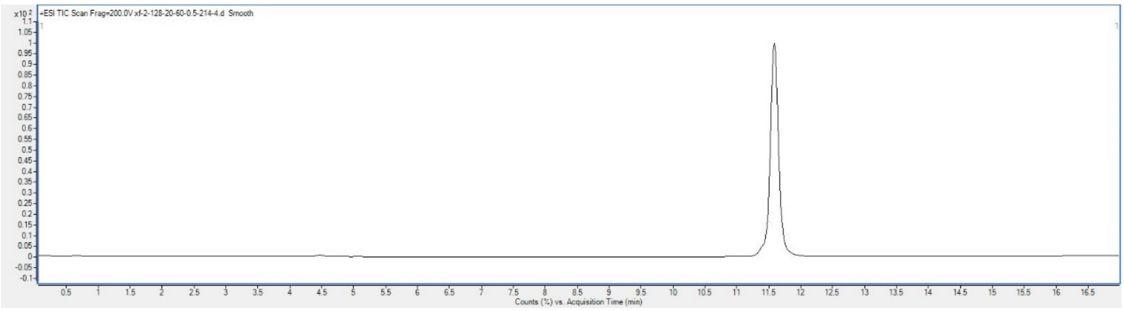

HRMS (ESI) m/z: $[M+H]^+$ Calcd for $C_{52}H_{84}N_{14}O_{21}S$ 1273.5729; Found 1273.5914.

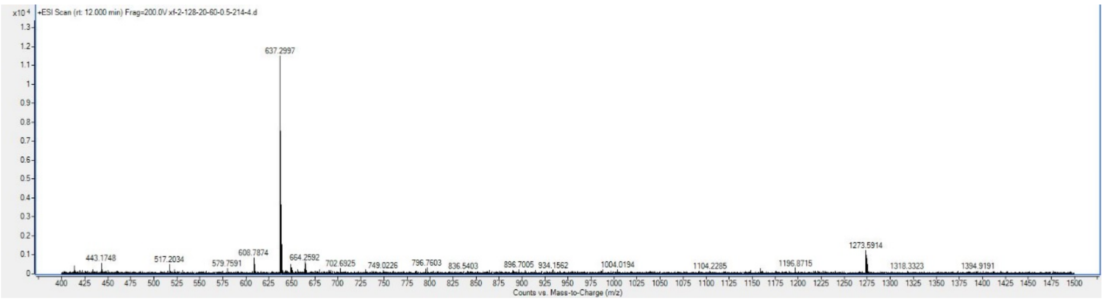

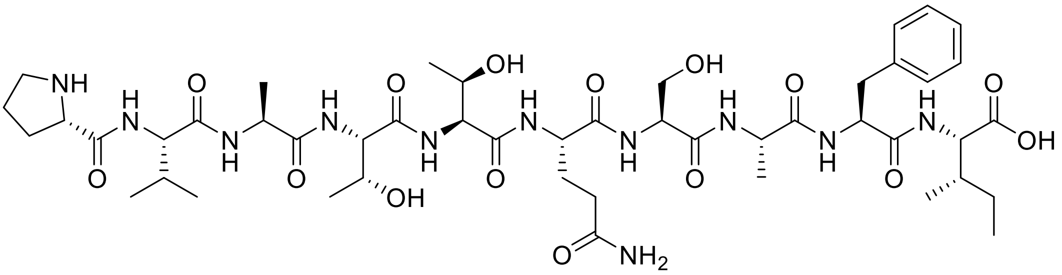

Chemical Formula: $C_{47}H_{75}N_{11}O_{15}$
Exact Mass: 1033.5444
21

Peptide **21** was prepared according to General Procedure D and E. 0.41 mg **3** (1.5 equiv.) and 0.50 mg **20** (1.0 equiv.) was dissolved in 200 μL Et_3N (pH = 10) solution. The resulting mixture was stirred for 6 h and the metal-free-desulfurization was finished in

1h. Purification of the crude peptide by preparative HPLC (20% to 60% solvent B over 20min, Higgins Analytical Proto 200 10 µm 250 × 20nm C18 column) afforded peptide **21** as a white solid after lyophilization (0.43 mg, 58%).

LC traces from crude reaction mixtures:

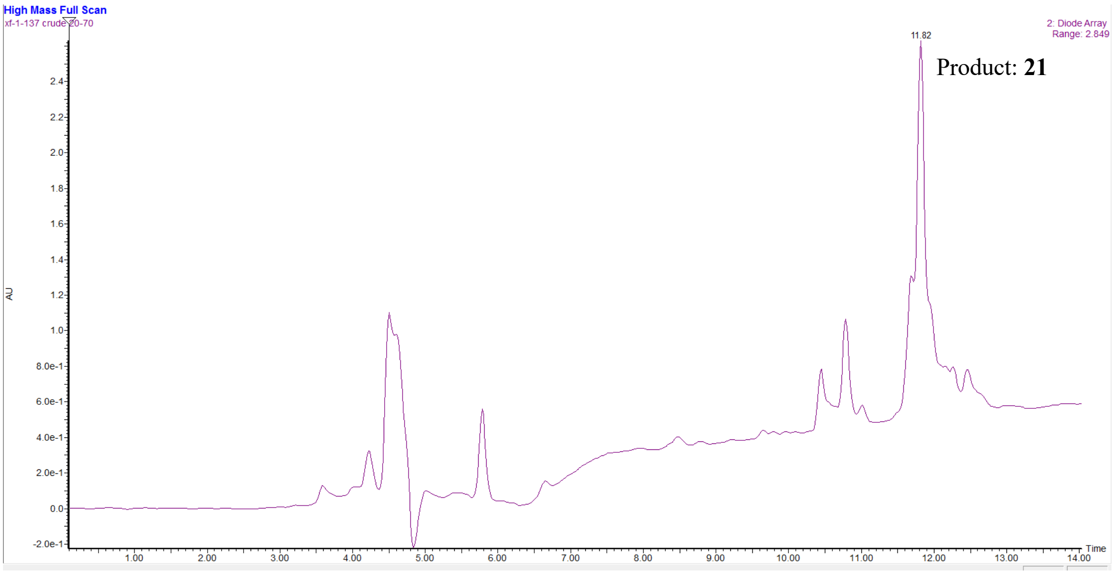

HPLC (Higgins Analytical Proto 200 5 µm 150 × 2.0 nm C18 column, water/acetonitrile = 80/20 to 30/70 over 20 min, flow rate = 0.5 mL/min, λ = 214 nm), tR= 10.73 min.

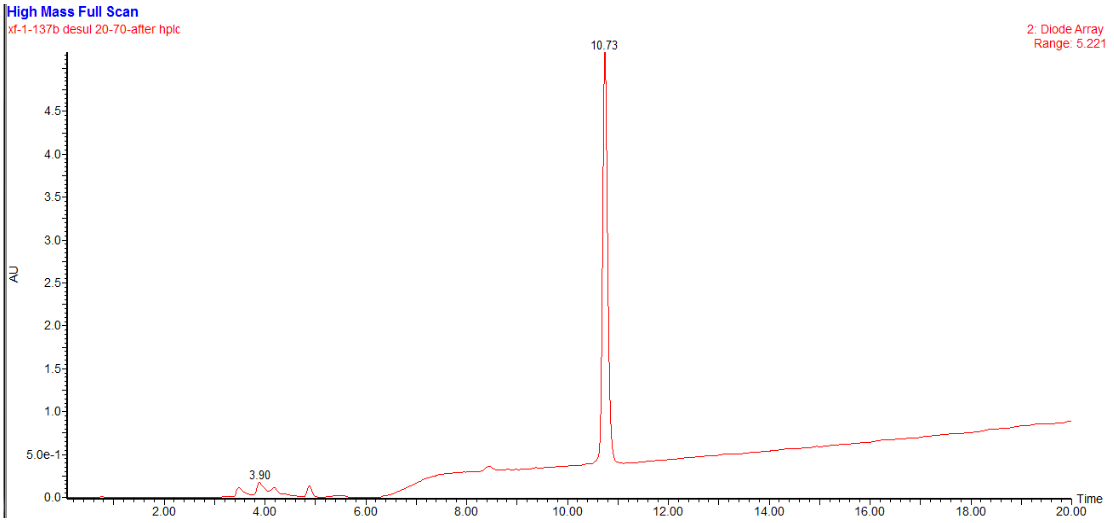

MS (ESI) m/z: $[M+H]^+$ Calcd for $C_{47}H_{75}N_{11}O_{15}$ 1034.5517; Found 1034.5350.

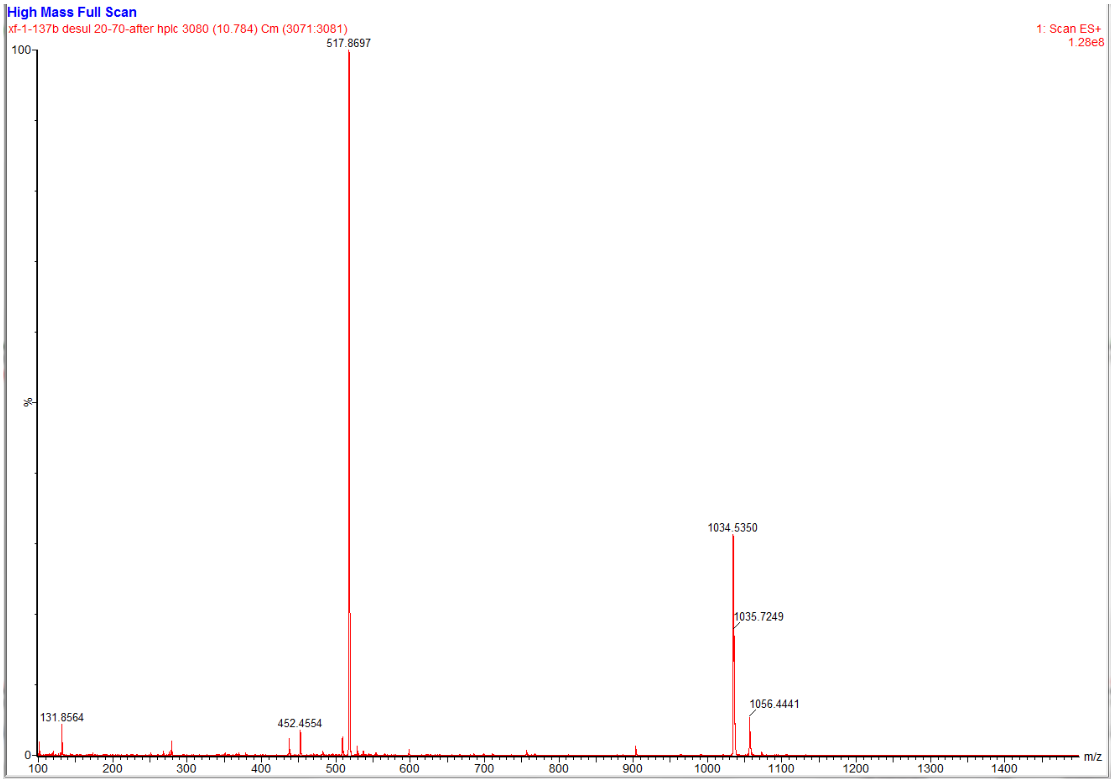

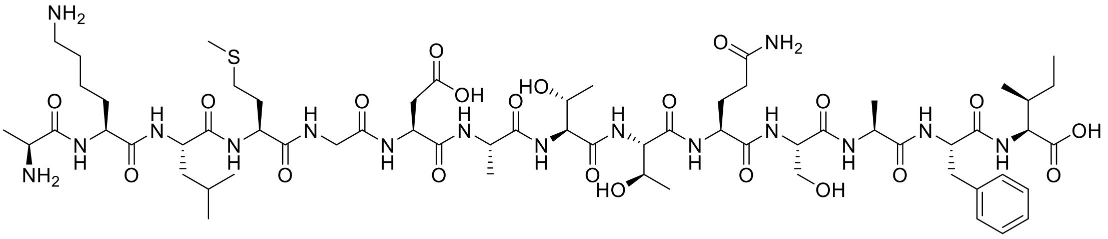

Chemical Formula: $C_{63}H_{104}N_{16}O_{21}S$
Exact Mass: 1452.7283
23

Peptide **23** was prepared according to General Procedure D and E. 0.91 mg **22** (1.5 equiv.) and 0.49 mg **20** (1.0 equiv.) was dissolved in 200 μL Et_3N (pH = 10) solution. The resulting mixture was stirred for 6 h and the metal-free-desulfurization was finished in 1h. Purification of the crude peptide by preparative HPLC (20% to 60% solvent B over 20min, Higgins Analytical Proto 200 10 μm 250 × 20nm C18 column) afforded peptide **23** as a white solid after lyophilization (0.33 mg, 32%).

LC traces from crude reaction mixtures:

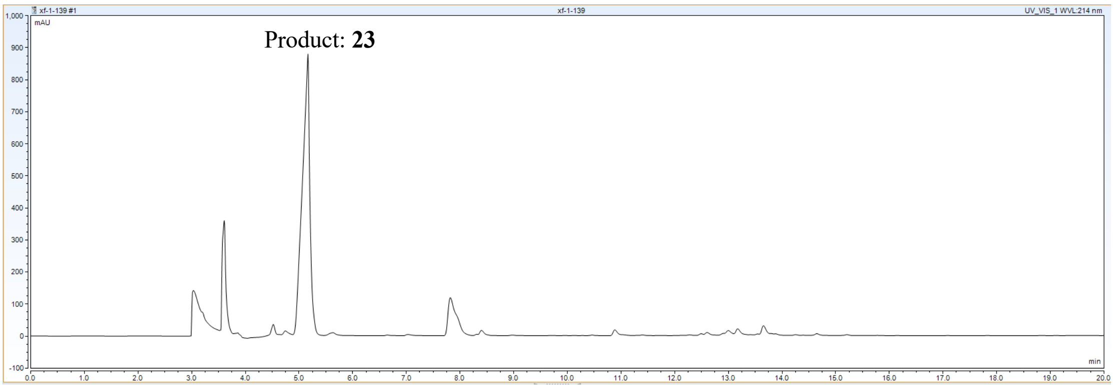

HPLC (Higgins Analytical Proto 200 5 µm 150 × 2.0 nm C18 column, water/acetonitrile = 80/20 to 40/60 over 20 min, flow rate = 0.5 mL/min, λ = 214 nm), tR= 11.20 min.

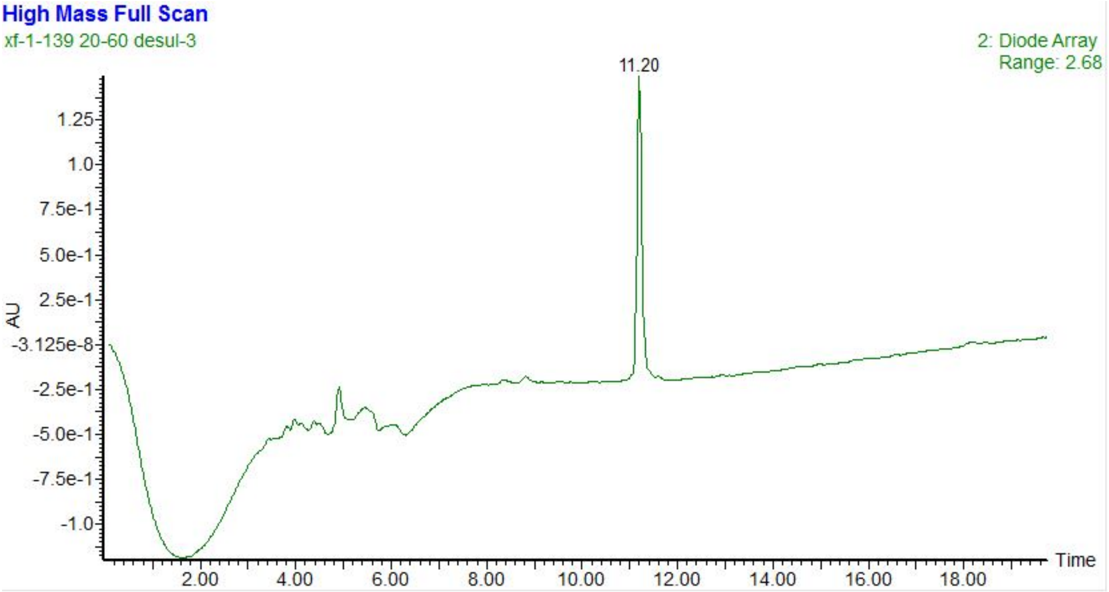

MS (ESI) m/z: $[M+H]^+$ Calcd for $C_{63}H_{104}N_{16}O_{21}S$ 1453.7355; Found 1453.9037.

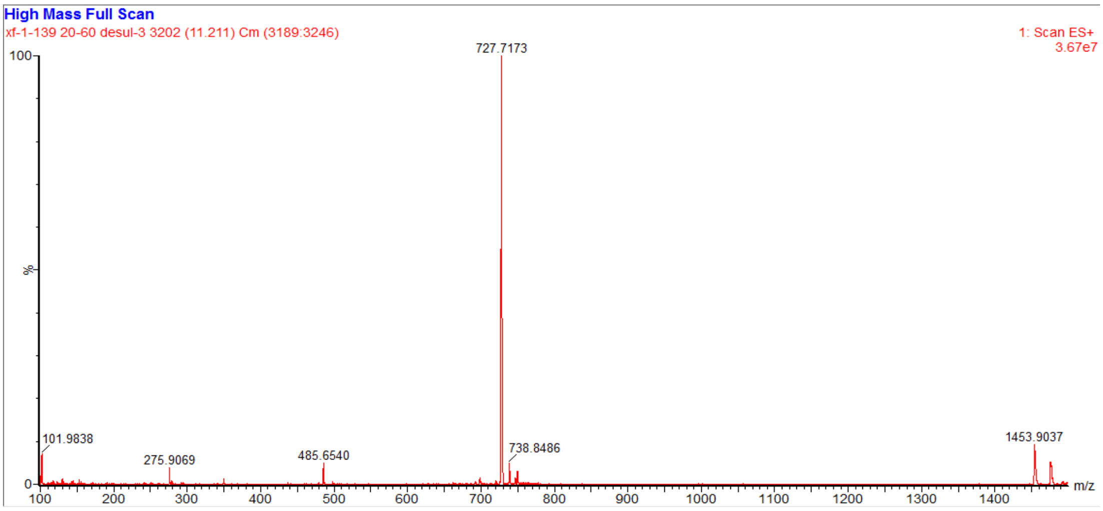

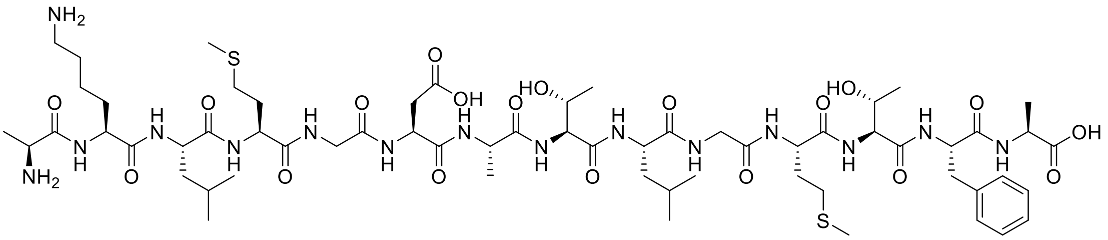

Chemical Formula: $C_{62}H_{103}N_{15}O_{19}S_2$
Exact Mass: 1425.6996
25

Peptide **25** was prepared according to General Procedure D and E. 0.92 mg **22** (1.5 equiv.) and 0.50 mg **24** (1.0 equiv.) was dissolved in 200 μL Et_3N (pH = 10) solution. The resulting mixture was stirred for 6 h and the metal-free-desulfurization was finished in 1h. Purification of the crude peptide by preparative HPLC (20% to 60% solvent B over 20min, Higgins Analytical Proto 200 10 μm 250 × 20nm C18 column) afforded peptide **25** as a white solid after lyophilization (0.40 mg, 38%).

LC traces from crude reaction mixtures:

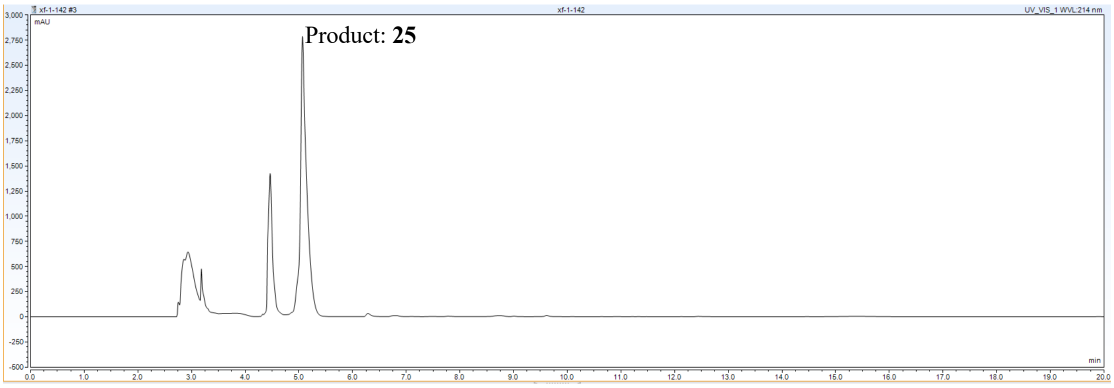

HPLC (Higgins Analytical Proto 200 5 μm 150 × 2.0 nm C18 column, water/acetonitrile = 80/20 to 40/60 over 20 min, flow rate = 0.5 mL/min, λ = 214 nm), tR= 12.78 min.

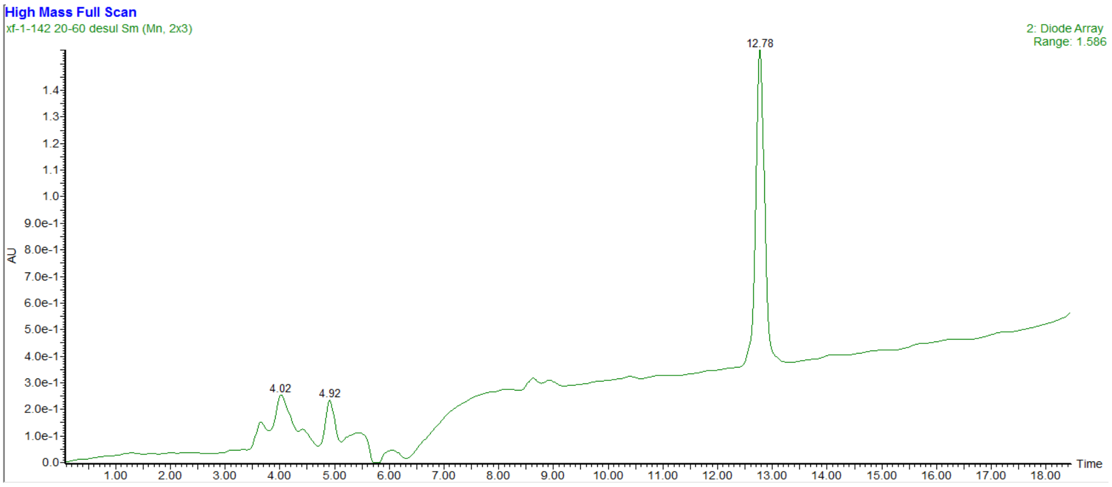

MS (ESI) m/z: $[M+H]^+$ Calcd for $C_{62}H_{103}N_{15}O_{19}S_2$ 1426.7069; Found 1426.6925.

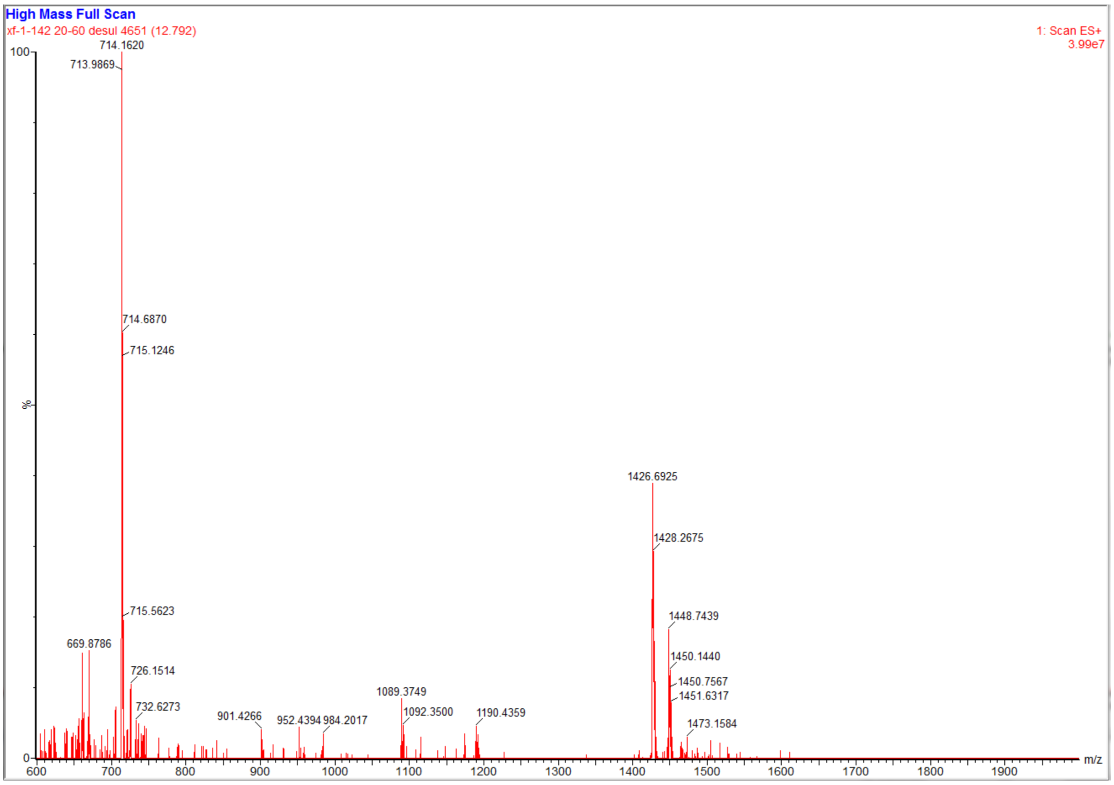

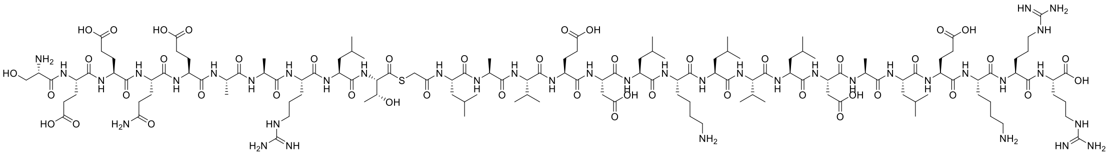

Chemical Formula: $C_{135}H_{233}N_{39}O_{46}S$
Exact Mass: 3168.6813
28

Thioester-peptide **28** was prepared according to General Procedure D. 0.41 mg **26** (1.5 equiv.) and 0.50 mg **27** (1.0 equiv.) was dissolved in 200 μL Et_3N (pH = 10) solution. The resulting mixture was stirred for 2 h. Then, purification of the crude peptide by preparative HPLC (20% to 60% solvent B over 20 min, Higgins Analytical Proto 200 10 μm 250 × 20nm C18 column) afforded peptide **28** as a white solid after lyophilization (0.59 mg, 76%).

LC traces from crude reaction mixtures:

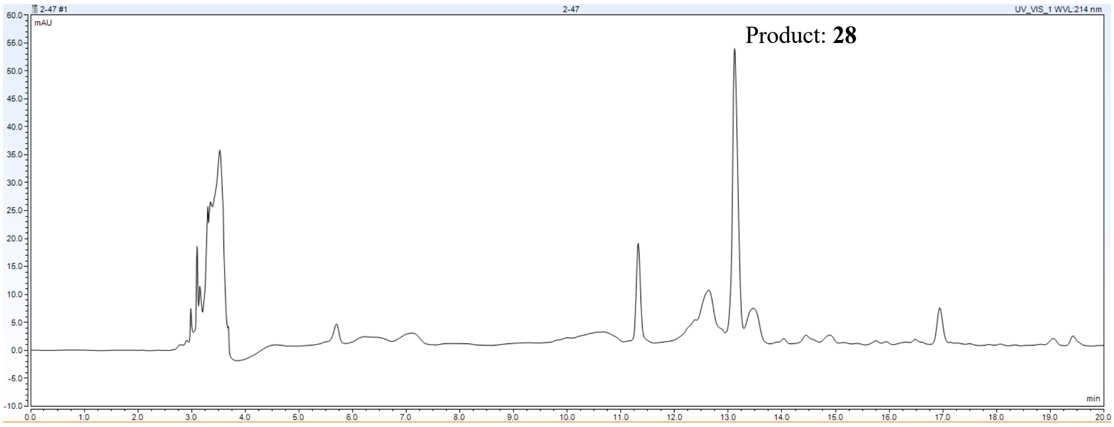

HPLC (Higgins Analytical Proto 200 5 μm 150 × 2.0 nm C18 column, water/acetonitrile = 80/20 to 30/70 over 20 min, flow rate = 0.5 mL/min, λ = 214 nm), tR= 14.58 min.

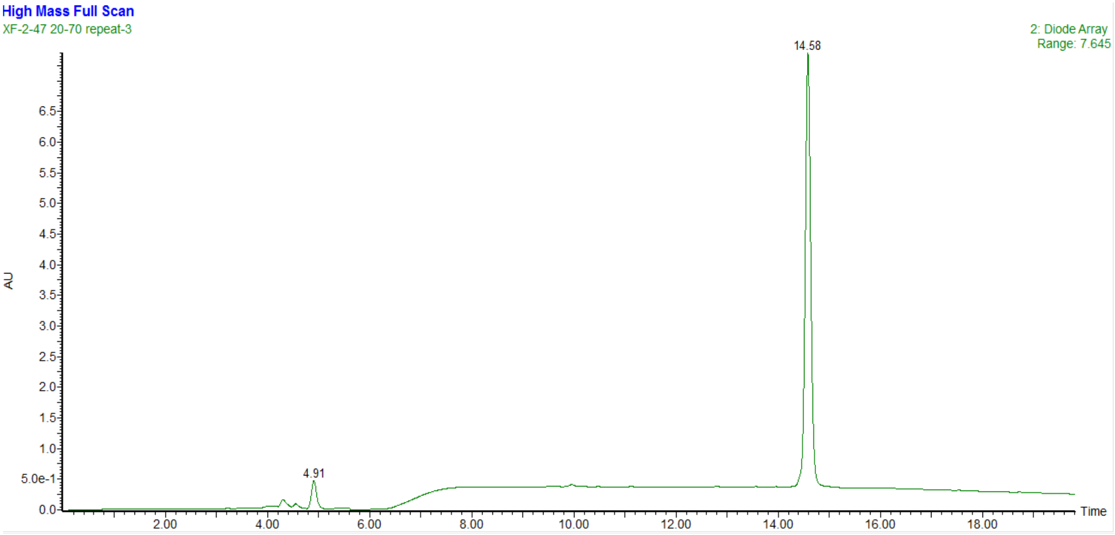

MS (ESI) m/z: [M+H]$^+$ Calcd for $C_{135}H_{233}N_{39}O_{46}S$ 3171.7031; Found 3172.6698.

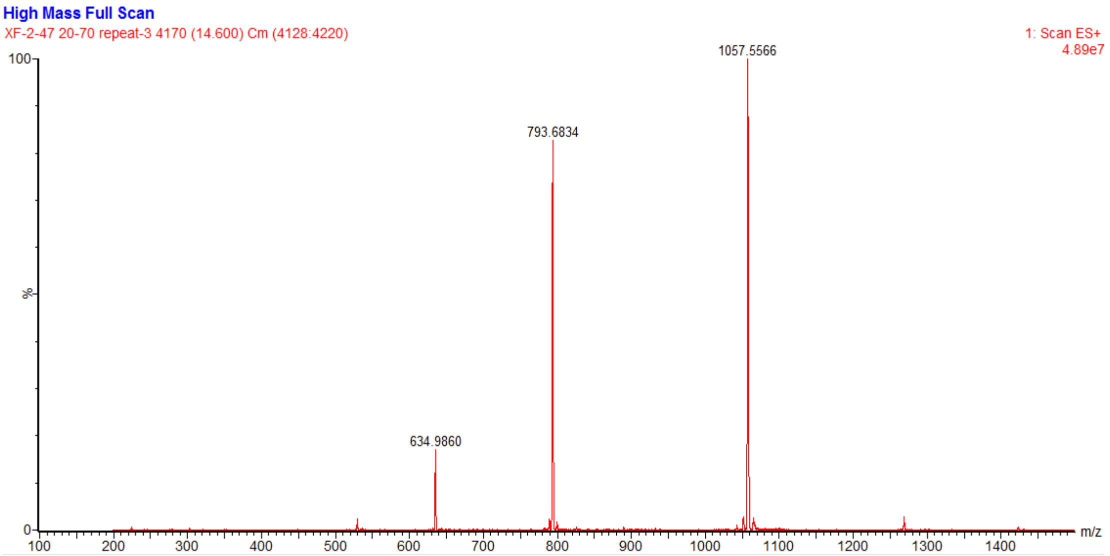

V Epimerization Free Experiment

To check the possible epimerization of NCL reaction between *β*-lactone mediated peptide and thiol-containing peptide, peptide Pro-Ala-Val-(L)-Thr-Cys-Val-Ala-Pro **29** (**5**) and Pro-Ala-Val-(D)-Thr-Cys-Val-Ala-Pro **30** was synthesized directly from SPPS method. By comparing the ^{1}H NMR of **29**, **30** and **5**, we could conclude there was no epimerization during NCL reaction.

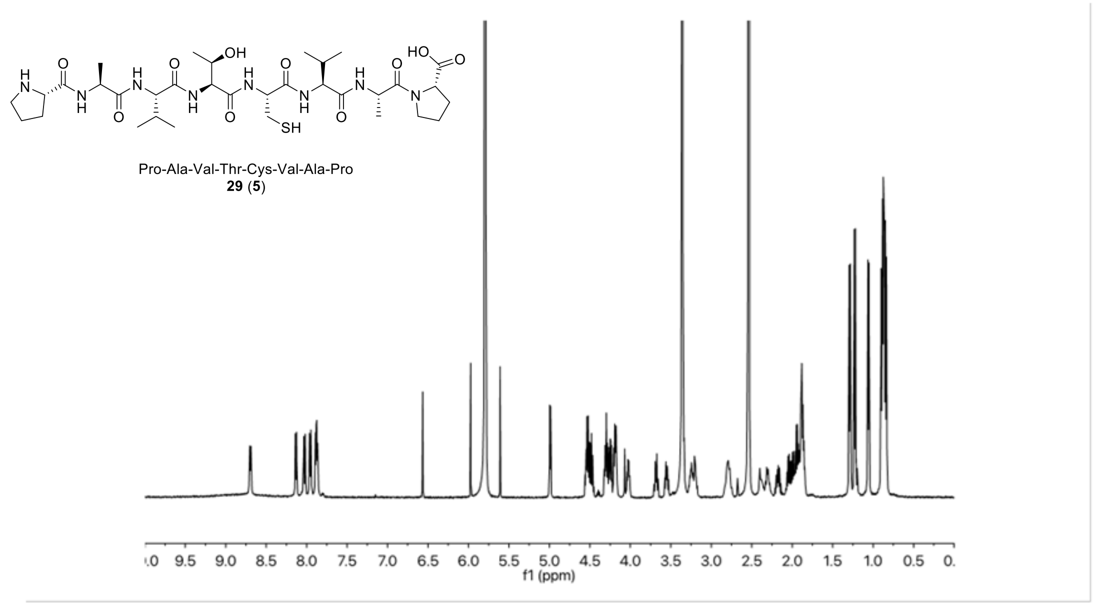
HO
O
OH
H
N
O
SH
Pro-Ala-Val-Thr-Cys-Val-Ala-Pro
29 (5)
9.5 9.0 8.5 8.0 7.5 7.0 6.5 6.0 5.5 5.0 4.5 4.0 3.5 3.0 2.5 2.0 1.5 1.0 0.5
f1 (ppm)

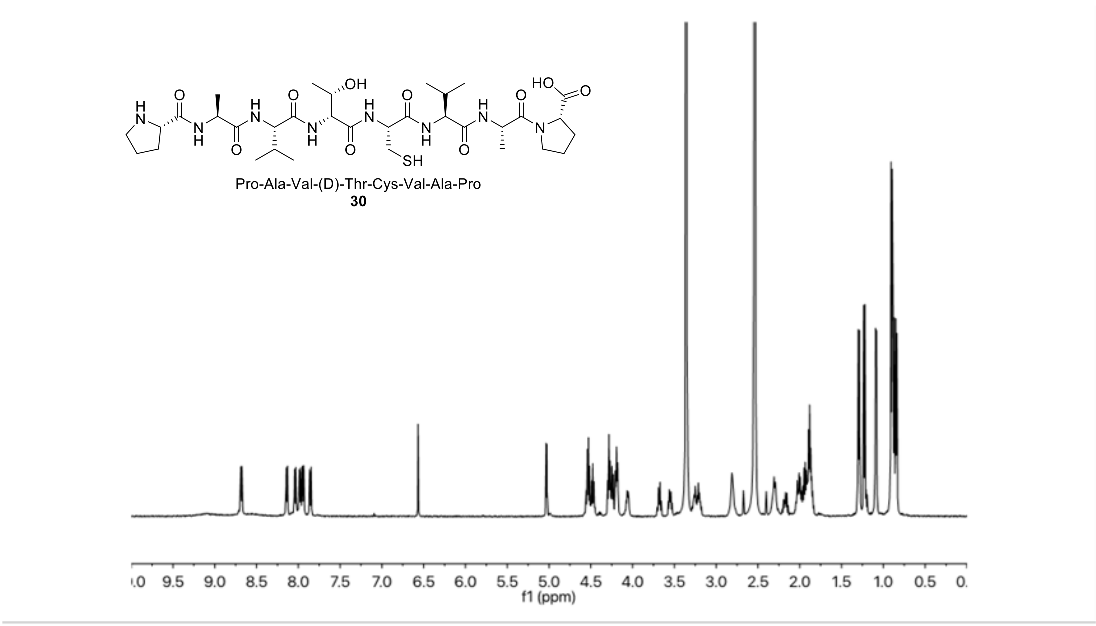
HO
O
OH
H
N
O
SH
Pro-Ala-Val-(D)-Thr-Cys-Val-Ala-Pro
30
9.5 9.0 8.5 8.0 7.5 7.0 6.5 6.0 5.5 5.0 4.5 4.0 3.5 3.0 2.5 2.0 1.5 1.0 0.5
f1 (ppm)

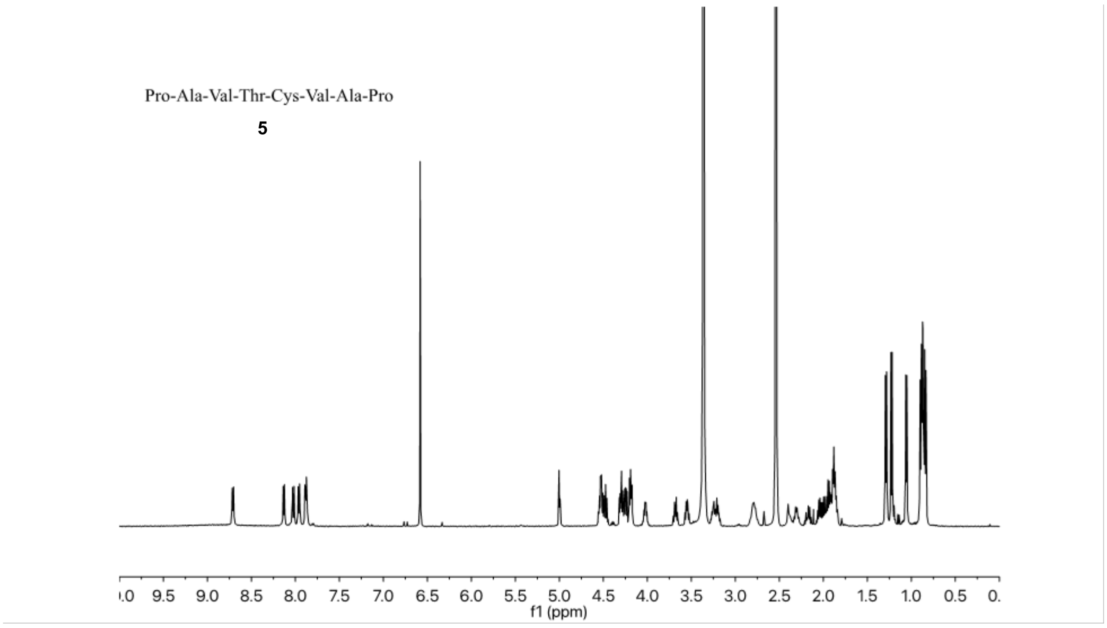

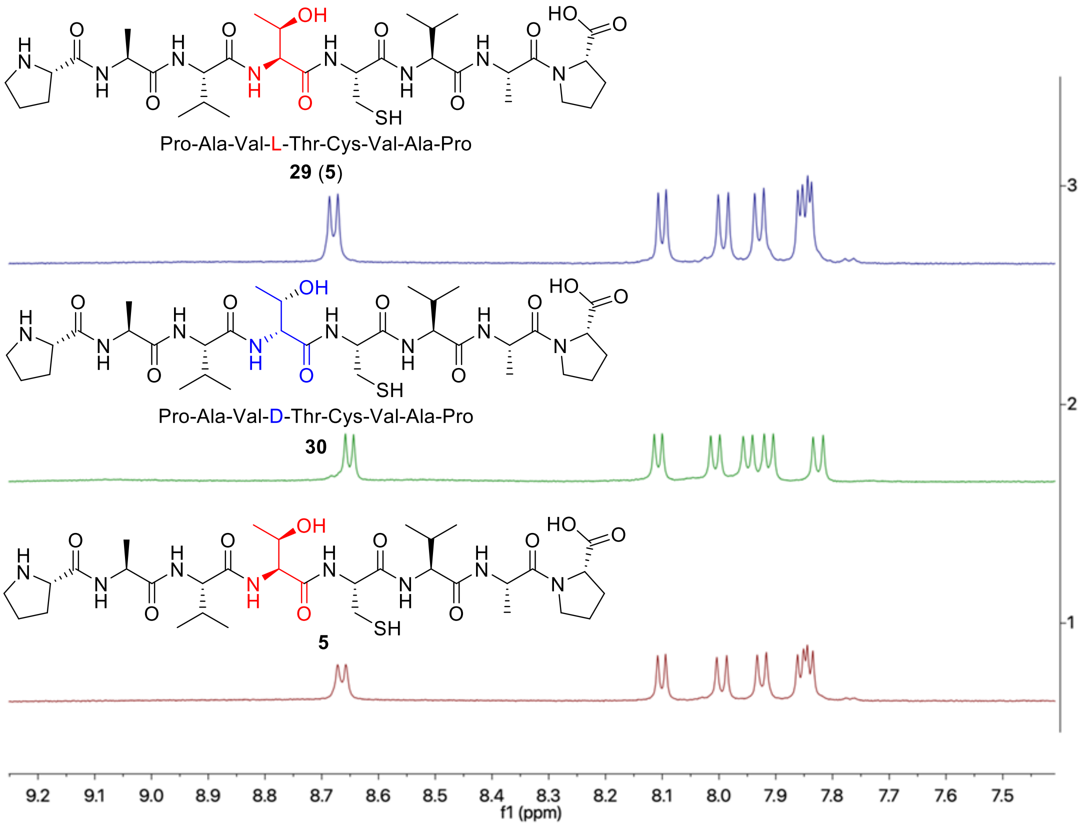

VI Four-component NCL Competition Experiment

Peptide **3** (0.5 mg, 1.0 equiv.), **31** (0.5 mg, 1.0 equiv.), **33** (0.5 mg, 1.0 equiv.) and **4** (0.5 mg, 1.0 equiv.) were dissolved in 200 μL Et_3N, pH=10 aqueous solution. The reaction was stirred at room temperature for 10 minutes. Check the product by LC-MS analysis.

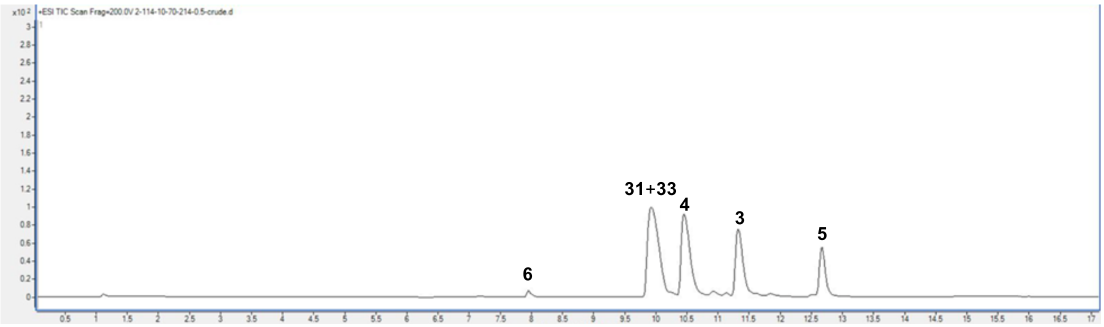

VII Cyclic Peptides Synthesis

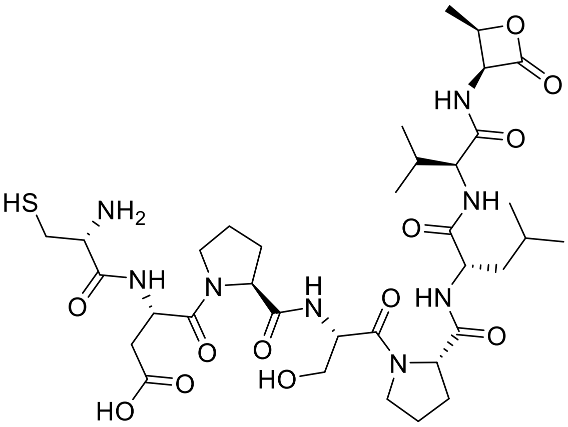

Chemical Formula: $C_{35}H_{56}N_8O_{12}S$
Exact Mass: 812.3738
35

According to the general procedure A and B, the heptapeptide Boc-Cys(Trt)-Asp(tBu)-Pro-Ser(tBu)- Pro-Leu-Val-OH was generated directly from SPPS on a 0.08 mmol scale. The resulting heptapeptide Boc-Cys(Trt)-Asp(tBu)-Pro-Ser(tBu)-Pro-Leu-Val-OH was coupled with β-lactone TFA salt **2** to afford the desired polypeptide following the general procedure C. Purification of the crude product using preparative HPLC (20 to 70% solvent B over 20 min, Higgins Analytical Proto 200 5 μm 250 × 10 nm C18 column) afforded peptide **35** as a white solid after lyophilization (8.2 mg, 31%).

HPLC (Higgins Analytical Proto 200 5 μm 150 × 2.0 nm C18 column, water/acetonitrile = 80/20 to 40/60 over 20 min, flow rate = 0.5 mL/min, λ = 214 nm), tR= 13.52 min.

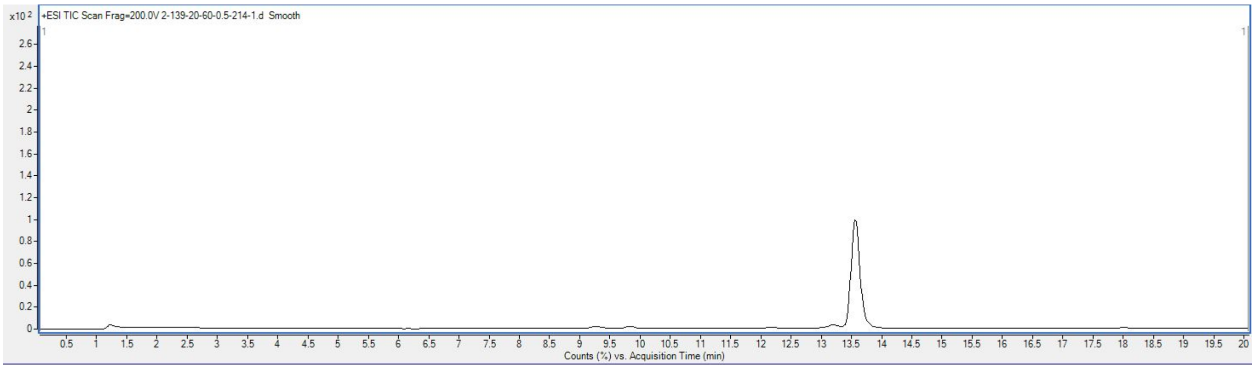

HRMS (ESI) m/z: $[M+H]^+$ Calcd for $C_{35}H_{56}N_8O_{12}S$ 813.3811; Found 813.4026.

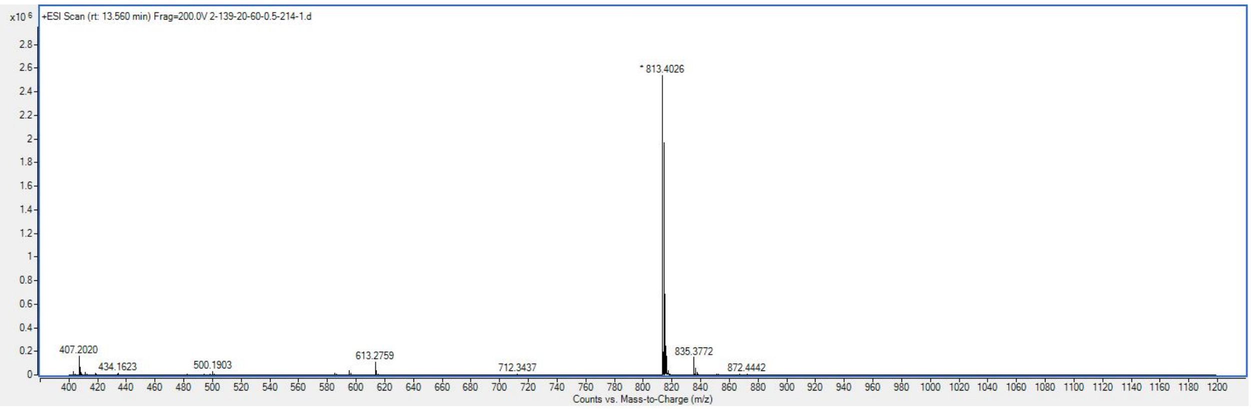

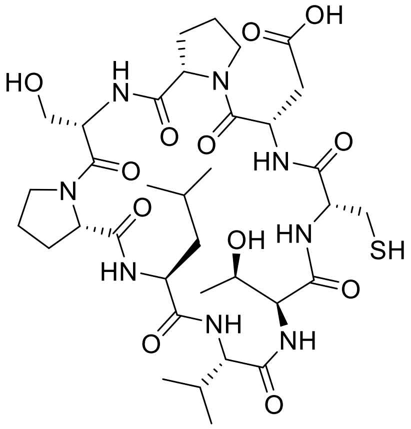

Chemical Formula: $C_{35}H_{56}N_8O_{12}S$
Exact Mass: 812.3738
36

Dissolve 1.0 mg peptide **35** in 200 µL Et_3N, pH=10 solution. Stir the mixture at room temperature for 2 hour. Purification of the crude product using preparative HPLC (20 to 70% solvent B over 20 min, Higgins Analytical Proto 200 5 µm 250 × 10 nm C18 column) afforded peptide **36** as a white solid after lyophilization (0.72 mg, 72%).

HPLC (Higgins Analytical Proto 200 5 µm 150 × 2.0 nm C18 column, water/acetonitrile = 80/20 to 40/60 over 20 min, flow rate = 0.5 mL/min, λ = 214 nm), tR= 14.57 min.

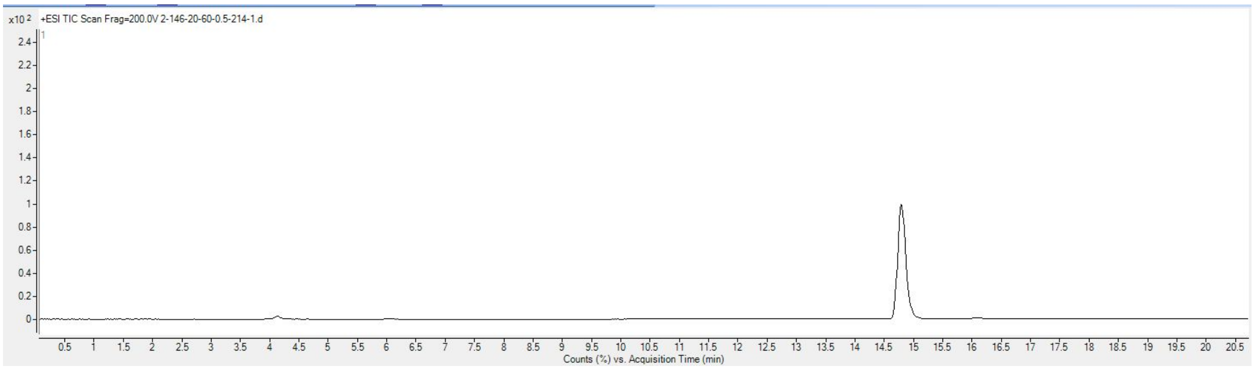

HRMS (ESI) m/z: $[M+H]^+$ Calcd for $C_{35}H_{56}N_8O_{12}S$ 813.3811; Found 813.3986.

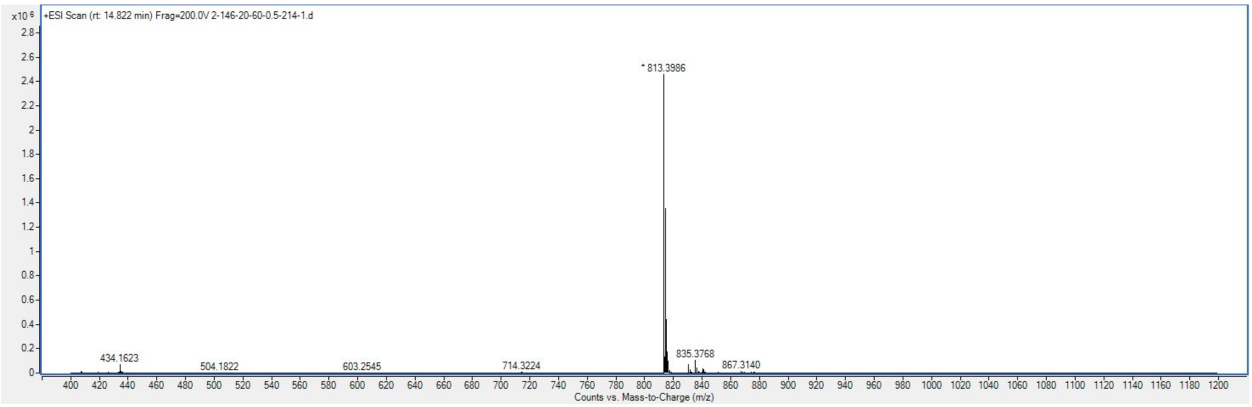

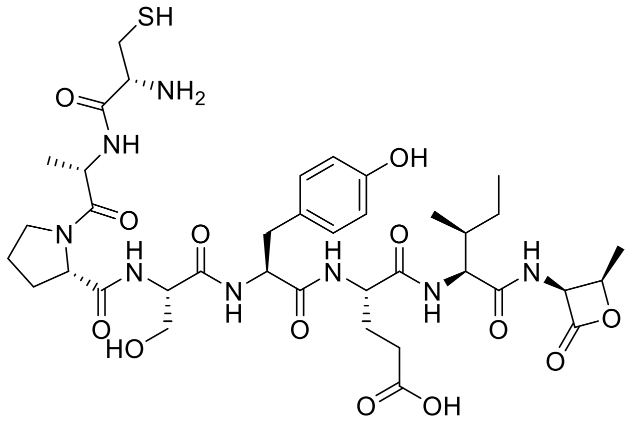

Chemical Formula: $C_{38}H_{56}N_8O_{13}S$
Exact Mass: 864.3688
37

According to the general procedure A and B, the heptapeptide Boc-Cys(Trt)-Ala-Pro-Ser(tBu)- Tyr(tBu)-Glu(tBu)-Ile-OH was generated directly from SPPS on a 0.08 mmol scale. The resulting heptapeptide Boc-Cys(Trt)-Ala-Pro-Ser(tBu)- Tyr(tBu)-Glu(tBu)-Ile-OH was coupled with β-lactone TFA salt **2** to afford the desired polypeptide following the general procedure C. Purification of the crude product using preparative HPLC (20 to 70% solvent B over 20 min, Higgins Analytical Proto 200 5 μm 250 × 10 nm C18 column) afforded peptide **37** as a white solid after lyophilization (5.2 mg, 22%).

HPLC (Higgins Analytical Proto 200 5 µm 150 × 2.0 nm C18 column, water/acetonitrile = 80/20 to 30/70 over 20 min, flow rate = 0.5 mL/min, λ = 214 nm), tR= 9.73 min.

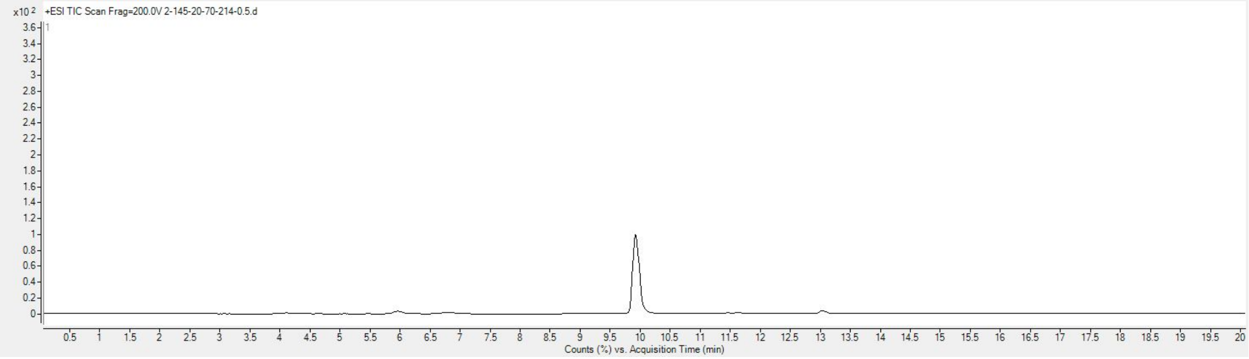

HRMS (ESI) m/z: $[M+H]^+$ Calcd for $C_{38}H_{56}N_8O_{13}S$ 865.3760; Found 865.3933.

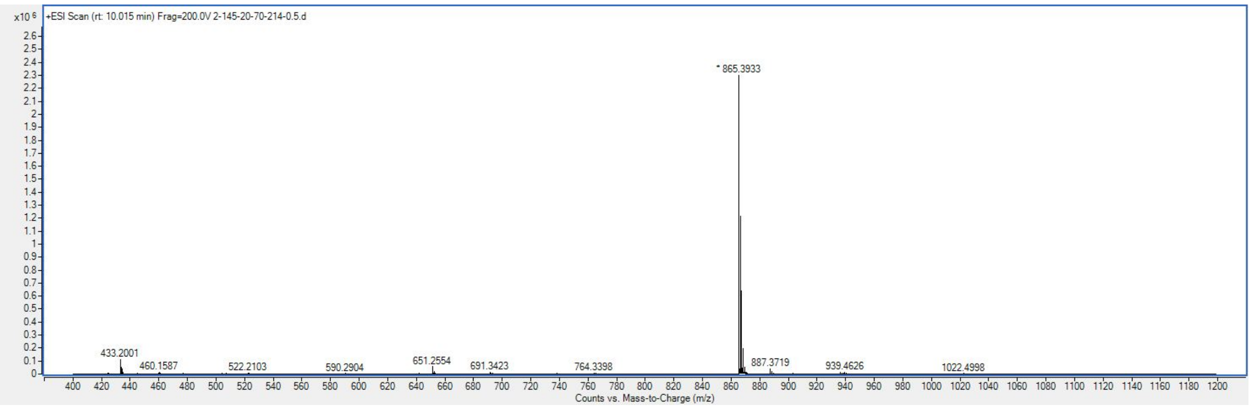

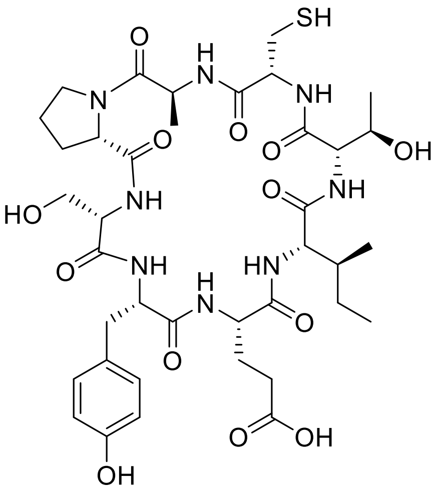

Chemical Formula: $C_{38}H_{56}N_8O_{13}S$

Exact Mass: 864.3688

38

Dissolve 1.0 mg peptide **37** in 200 µL Et_3N, pH=10 solution. Stir the mixture at room temperature for 2 hour. Purification of the crude product using preparative HPLC (20 to 70% solvent B over 20 min, Higgins Analytical Proto 200 5 µm 250 × 10 nm C18 column) afforded peptide **38** as a white solid after lyophilization (0.63 mg, 63%).

HPLC (Higgins Analytical Proto 200 5 µm 150 × 2.0 nm C18 column, water/acetonitrile = 80/20 to 30/70 over 20 min, flow rate = 0.5 mL/min, λ = 214 nm), tR= 13.16 min.

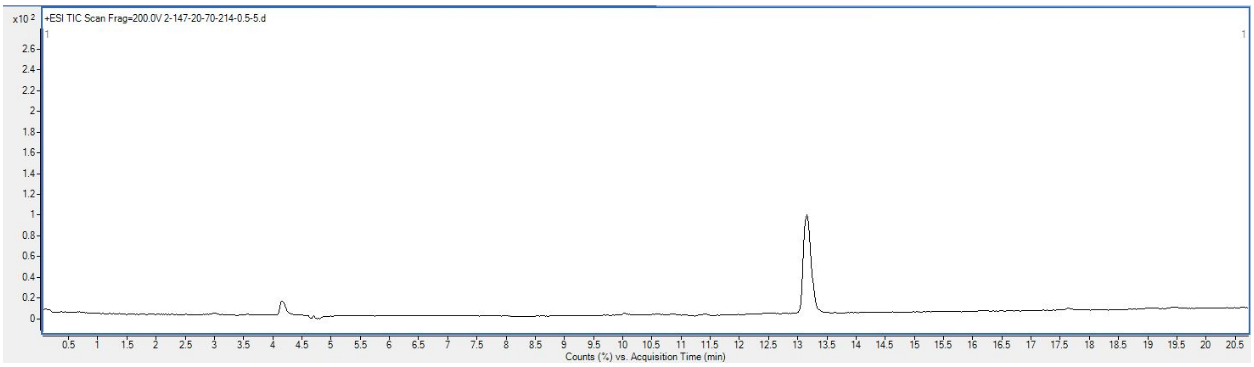

HRMS (ESI) m/z: $[M+H]^+$ Calcd for $C_{38}H_{56}N_8O_{13}S$ 865.3760; Found 865.3889.

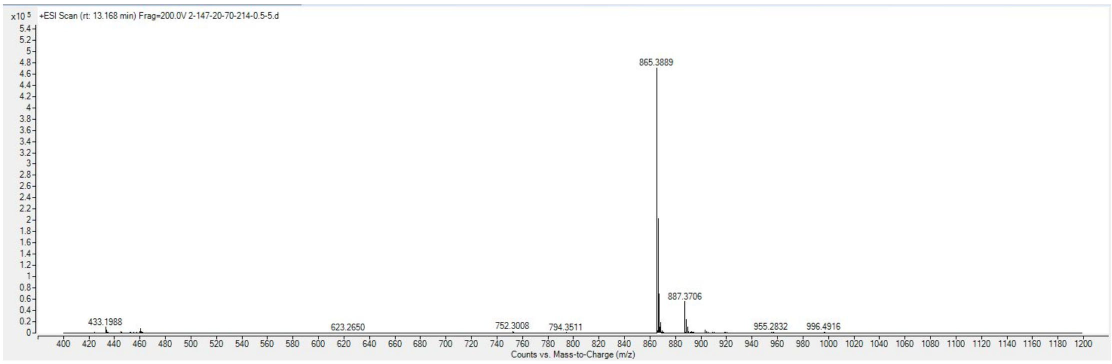

VIII Reference:

(1) Wang, Z.; Gu, C.; Colby, T.; Shindo, T.; Balamurugan, R.; Waldmann, H.; Kaiser, M.; van der Hoorn, R. A. L., Β-Lactone Probes Identify a Papain-Like Peptide Ligase in Arabidopsis Thaliana. *Nat. Chem. Biol.* **2008**, *4* (9), 557-563.
